# Supplementary material for: Energy transfer-mediated multiphoton synergistic excitation for selective C(sp3)–H functionalization with coordination polymer
Source: Nat Commun. 2024 Oct 11;15:8813. doi: 10.1038/s41467-024-53115-6 (PMC11470074; doi:10.1038/s41467-024-53115-6)
Supplement: Supplementary file 1 — Supplementary Information [file 41467_2024_53115_MOESM1_ESM.pdf]

<Supplementary information>

Energy transfer-mediated multiphoton synergistic excitation for selective C(sp^3)−H functionalization with coordination polymer

Zhonghe Wang,[1,3] Yang Tang,[1,3] Songtao Liu,[1,3] Liang Zhao,[1,*] Huaqing Li,[1] Cheng He,[1] and Chunying Duan[1,2,*]

[1]State Key Laboratory of Fine Chemicals, Frontier Science Center for Smart Materials, School of Chemical Engineering, Dalian University of Technology, Dalian 116024, People's Republic of China

[2]State Key Laboratory of Coordination Chemistry, Nanjing University, Nanjing 210093, People's Republic of China

[3]These authors contributed equally: Zhonghe Wang, Yang Tang, Songtao Liu.

Correspondence to: zhaol@dlut.edu.cn; cyduan@dlut.edu.cn

Table of Contents

1. Materials and Methods
2. Preparation of Metal–Organic Frameworks
3. Single Crystal X-ray Crystallography
4. Characterization of Metal–Organic Frameworks
5. Data Relative to Photocatalytic Reaction
6. DFT Calculations
7. The $^1H/^{13}C$ NMR Spectra of C(*sp*^{3})–H Alkylation Products
8. The $^1H/^{13}C$ NMR of Decarboxylative Functionalization Products
9. Quantification Method of Oxidation Product by Gas Chromatography
10. References

1. Materials and Methods

All the chemicals and solvents were of reagent grade quality obtained from commercial sources and used without further purification. The ligand *N*,*N*'-bis(5-isophthalic acid)naphthalenediimide (H_4BINDI) was synthesized according to the literature.[1] The elemental analyses of C, H and N were performed on an Elementar UNICUBE elemental analyzer. ^1H / ^{13}C NMR spectra were recorded by Vaian DLG400 with internal standard TMS at δ 0.0 ppm. ESI-MS measurements were performed on an Agilent 6224 HPLC-TOF spectrometer. Powder X-ray diffraction (PXRD) measurements were obtained on a Rigaku Smart Lab XRD instrument with a sealed Cu tube (λ = 1.54178 Å). Thermogravimetric analyses were performed on a TA Q500 instrument and recorded under N_2 followed by a ramp of 10 °C·min^{-1} up to 800 °C. Fourier transform infrared spectroscopy spectra were recorded using KBr pellets on a ThermoFisher 6700. Energy-dispersive system elemental mapping images were obtained on a JEOL JSM-7610F Plus Field Emission Scanning Electron Microscopy. Liquid UV-Vis spectra were collected on a PERSEE T9CS spectrometer. Solid UV-vis spectra were recorded on Hitachi UH5700 UV-vis-NIR spectrophotometer. Fluorescent spectra were recorded on Edinburgh FLS 1000 stable/transient fluorescence spectrometer. The EPR spectra were performed on BRUKER E500 equipped with a liquid N_2 system. X-ray photoelectron spectroscopy (XPS) signals were collected on a Thermo ESCALAB Xi+ spectrometer. The light source is 455 nm LED with a half peak width of 30 nm, which was purchased from the Beijing China Education Au-light Co. Ltd. The gas chromatography-mass spectrometry (GC-MS) analyses were performed on Agilent Technologies 7890B GC system and Agilent 5977B MSD system. ^{57}Fe Mössbauer spectroscopy were recorded on a conventional spectrometer with alternating constant acceleration of the γ-source (57Co/Rh, 0.925 GBq), which was kept at room temperature. The minimum experimental line width was 0.24 mm s^{-1} (full width at half-height).

1.1 Electrochemical experiment

Solid-state cyclic voltammogram was measured on ZAHNER ENNIUM electrochemical workstation by using a three-electrode system with an Ag/AgCl electrode as a reference electrode, a platinum silk with 0.5 mm diameter as a counter electrode, and a homemade carbon-paste electrode as a working electrode in an aqueous solution of KCl at a scan rate of 100 $mV \cdot s^{-1}$. The manufacturing operation of carbon-paste working electrode: a well-ground mixture of sample and carbon paste (graphite and moderate mineral oil) was set in the channel of a glass tube and connected to a copper wire.

1.2 General procedure for the C(sp^3)−H alkylation

A 20 mL of flame-dried Schlenk quartz flask was added Fe−NDI (5.0 μmol), Benzylidene malononitrile (0.1 mmol), cyclohexane (1.0 mmol) and HCl (0.05 mmol concentrated HCl) in CH_3CN (1 mL). The resulting mixture was stirred and irradiated with a 455 nm LED under argon atmosphere at room temperature for 12 hours. After the indicated time, the mixture was centrifuged at 2500 g for 5 min, and the supernatant was concentrated under vacuum distillation. The residues were separated on a silica gel column (EtOAc/petroleum ether) to obtain the isolated yields.

1.3 General procedure for the ethane, propane and butane alkylation

A 20 mL of flame-dried Schlenk quartz flask was added Fe−NDI (10.0 μmol), Benzylidene malononitrile (0.2 mmol) and HCl (0.10 mmol concentrated HCl) in CH_3CN (7 mL). The resulting mixture was stirred and irradiated with a 455 nm LED and the reaction mixture was carried out with balloon with light alkane at room temperature for 48 hours. After the indicated time, the mixture was centrifuged at 2500 g for 5 min, and the supernatant was concentrated under vacuum distillation. The residues were separated on a silica gel column (EtOAc/petroleum ether) to obtain the isolated yields.

1.4 General procedure for the methane alkylation

The reaction was performed with benzylidene malononitrile (0.1 mmol), Fe–NDI (15.0 μmol), methane (5 MPa), HCl (0.10 mmol concentrated HCl) in CH_3CN (7 mL) under 455 nm LED irradiation for 48 hours. After the indicated time, the mixture was centrifuged at 2500 g for 5 min, and the supernatant was concentrated under vacuum distillation. The residues were separated on a silica gel column (EtOAc/petroleum ether) to obtain the isolated yields.

1.5 General procedure for the decarboxylative functionalization

A 20 mL of flame-dried Schlenk quartz flask was added Fe−NDI (5.0 μmol), Benzylidene malononitrile (0.1 mmol) and cyclohexanecarboxylic acid (0.50 mmol) in 1,4-dioxane (1 mL). The resulting mixture was stirred and irradiated with a 455 nm LED under argon atmosphere at room temperature for 12 hours. After the indicated time, the mixture was centrifuged at 2500 g for 5 min, and the supernatant was concentrated under vacuum distillation. The residues were separated on a silica gel column (EtOAc/petroleum ether) to obtain the isolated yields.

1.6 General procedure for the oxidation reaction

A 20 mL of flame-dried Schlenk quartz flask was added Fe−NDI (10.0 μmol), alkane (0.2 mmol), NH_4Cl (0.05 mmol) in CH_3CN (1.0 mL). The resulting mixture was stirred and irradiated with a 455 nm LED under air atmosphere at room temperature for 12 hours. After the indicated time, the mixtures were filtered and the yields were determined by gas chromatography.

1.7 Reactive radical scavenging experiments

A series of radical scavengers were employed to to investigate the role of each radical in the process of oxidation reaction. i.e., t-BuOH as the scavenger of hydroxyl radicals ($^{\bullet}OH$), catalase (H_2O_2), p-benzoquinone as the scavenger of $O_2^{\bullet-}$, NaN_3 and TEMPO as the scavenger of $^1O_2$ and alkyl radicals respectively. The corresponding controlled reactions were performed similarly to the above photocatalytic procedure where the radical scavengers (0.2 mmol) or catalase (30000 U) were added to the standard reaction system.

2. Preparation of Metal−Organic Frameworks

Synthesis of Fe−NDI

H_4BINDI (0.1 mmol) and $FeCl_3 \cdot 6H_2O$ (0.2 mmol) were added into high pressure reactor. The mixture added DMF (5 mL), acetic acid (0.1 mL) and H_2O (0.1 mL), then the reactor was ultrasound for ten minutes. The reactor was gradually heat up to 120 ºC and within three days. After self-assembly finished, the reactor slowly cooled to room temperature. The layered yellow crystal was collected by filtration and drying. The yield of Fe−NDI was 35% based on BINDI. Anal. Calcd (%) for Fe−NDI ($C_{21}H_{19}FeN_3O_8$): C, 50.68; H, 3.85; N, 8.45. Found: C, 50.26; H, 3.89; N, 8.31. IR (KBr): 3083 (br, v), 1712 (vs), 1681 (m), 1586 (m), 1452 (w), 1407 (w), 1351 (s), 1284

(w), 1252 (s), 1204 (w), 1169 (w), 1122 (m), 990 (w), 767 (s), 738 (s), 683 (w), 652 (s), 542 (w), 572 (w), 415 (m) cm^{-1}.

3. Single Crystal X-ray Crystallography

Intensities of Fe−NDI was collected on a Bruker SMART APEX CCD diffractometer equipped with a graphite-monochromated Mo-Kα (λ = 0.71073 Å) radiation source; the data were acquired using the SMART and SAINT programs.[2,3] The structures were solved by direct methods and refined on F^2 by full-matrix least-squares methods using the SHELXTL version 5.1 software.[4] In the structural refinement of Fe−NDI, all the non-hydrogen atoms were refined anisotropically. Hydrogen atoms within the ligand backbones and the coordinate DMF molecules were fixed geometrically at calculated distances and allowed to ride on the parent non-hydrogen atoms. The SQUEEZE subroutine in PLATON was used.[5]

Table S1. Crystal data and structure refinements.

Compound	Fe–NDI
Empirical formula	$C_{21}H_{19}FeN_3O_8$
Formula weight	497.24
T / K	250.0
Crystal system	Monoclinic
Space group	*P2(1)/c*
a / Å	20.133(5)
b / Å	16.304(4)
c / Å	10.135(2)
α / °	90
β / °	103.079(5)
γ / °	90
V / Å^3	3240.7(13)
Z	4
D_{calcd} / g cm^{-3}	1.019
μ / mm^{-1}	0.501
F (000)	1024
Refl.	39815 / 7610
collected / unique	[R_{int} = 0.0611]
Data / restraints / parameters	7610 / 48 / 309
Goodness-of-fit on F^2	1.062
$R[I>2\sigma(I)]^a$	R_1 = 0.0700
	wR_2 = 0.2039
R indices (all data)^b	R_1 = 0.0867
	wR_2 = 0.2140
$\Delta\rho_{max,min}$ / eÅ^{-3}	0.762 / -0.512
CCDC number	2282498

$^aR_1 = \Sigma||F_o| - |F_c||/\Sigma|F_o|$; $^bwR_2 = \Sigma[w(F_o^2 - F_c^2)^2]/\Sigma[w(F_o^2)^2]^{1/2}$

Figure S1. Single-crystal X-ray diffraction structure of Fe–NDI asymmetric unit. Iron = green, Oxygen = red, Nitrogen = blue, Carbon = grey, Hydrogen = white.

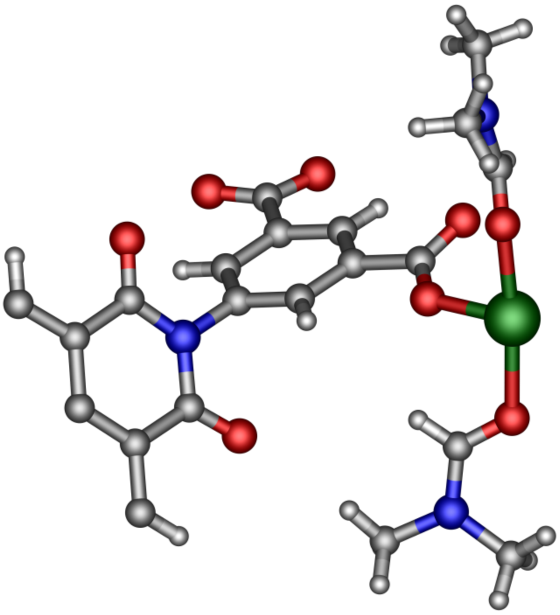

Figure S2. Parallelogram channels contained in layers for Fe−NDI.

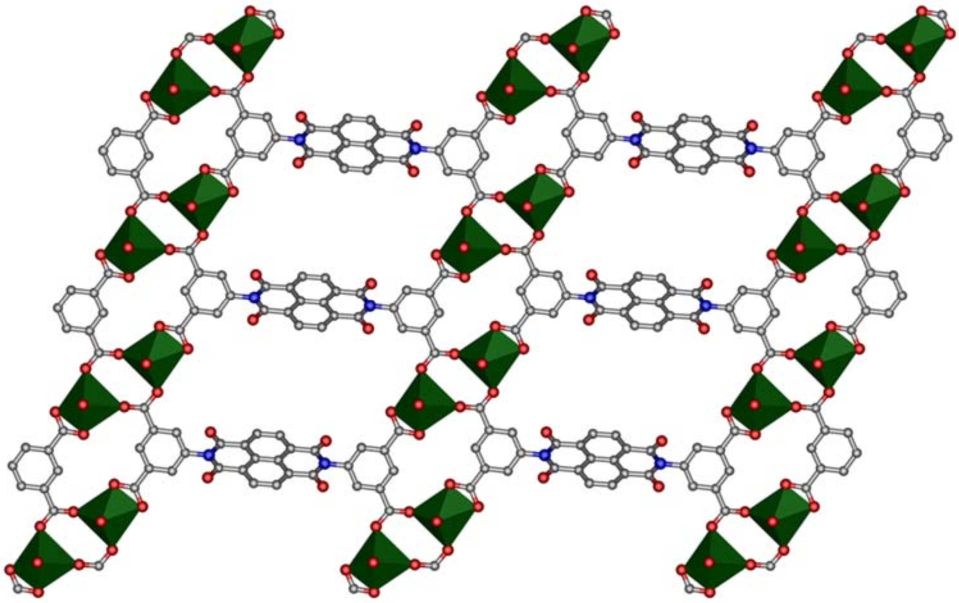

Figure S3. The hydrogen bond existed between layers.

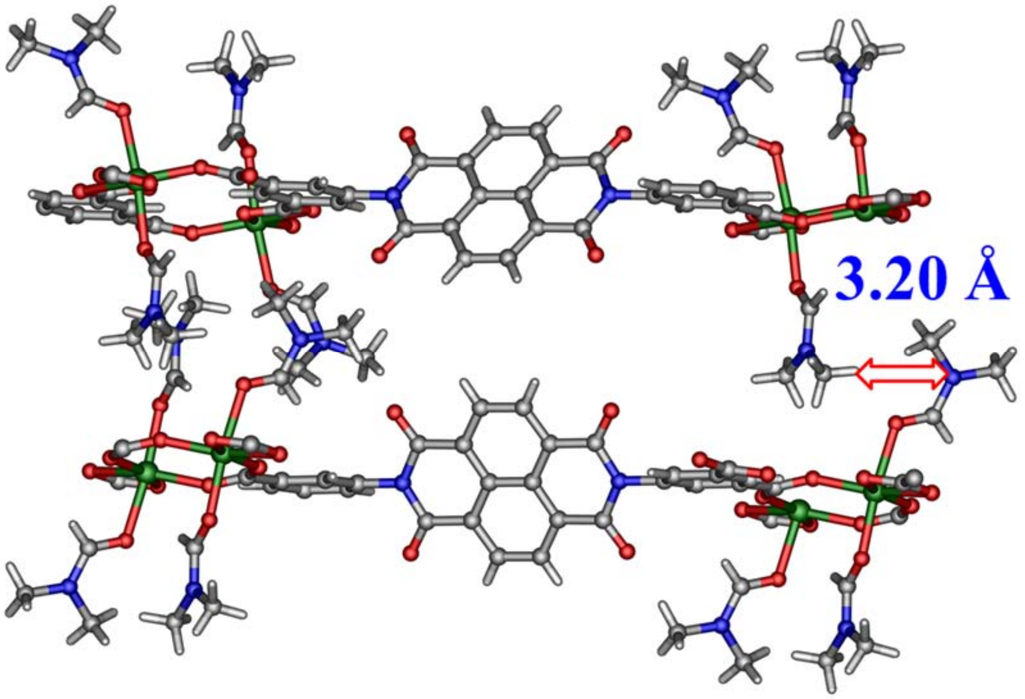

Figure S4. The coordinated environments and the polyhedral representation of binuclear [Fe2].

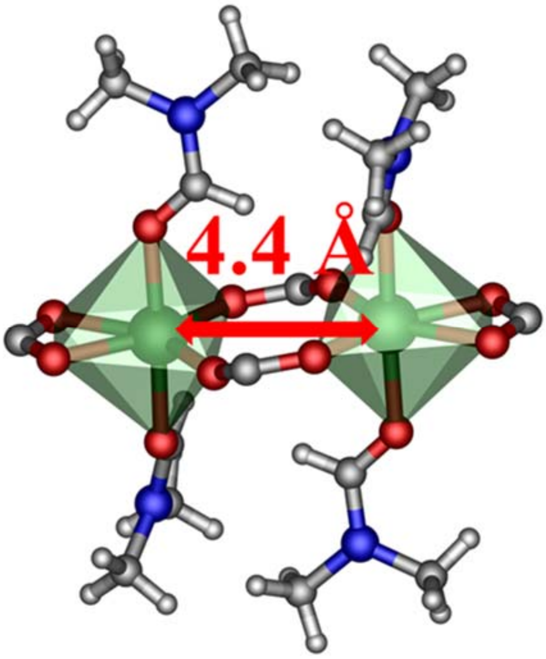

Table S2. Selected bond lengths (Å) for Fe−NDI.

Bond	Length/Å	Bond	Length/Å
Fe(1)-O(1)	2.024(2)	Fe(1)-O(3)#2	2.079(2)
Fe(1)-O(2)#1	2.043(2)	Fe(1)-O(4)#2	2.321(3)
Fe(1)-O(21)	2.132(3)	Fe(1)-O(31)	2.196(3)
O(2)-Fe(1)#1	2.043(2)	O(3)-Fe(1)#3	2.079(2)
O(4)-Fe(1)#3	2.321(3)	N(1)-C(7)	1.452(4)
C(1)-C(3)	1.497(4)	O(1)-C(1)	1.260(4)

Table S3. Selected bond angles (°) for Fe−NDI.

Bond	Angle/°	Bond	Angle/°
O(1)-Fe(1)-O(2)#1	108.31(10)	O(1)-Fe(1)-O(3)#2	155.72(10)
O(1)-Fe(1)-O(4)#2	96.67(10)	O(1)-Fe(1)-O(21)	91.06(11)
O(1)-Fe(1)-O(31)	86.24(11)	O(2)#1-Fe(1)-O(3)#2	95.83(9)
O(2)#1-Fe(1)-O(4)#2	154.57(10)	O(2)#1-Fe(1)-O(21)	93.03(12)
O(2)#1-Fe(1)-O(31)	92.87(11)	O(3)#2-Fe(1)-O(4)#2	59.06(9)
O(3)#2-Fe(1)-O(21)	90.20(11)	O(3)#2-Fe(1)-O(31)	90.09(10)
O(21)-Fe(1)-O(4)#2	91.16(11)	O(21)-Fe(1)-O(31)	174.03(11)
O(31)-Fe(1)-O(4)#2	83.88(10)	C(9)-N(1)-C(7)	117.6(3)
C(13)-N(1)-C(9)	124.8(3)	C(1)-O(1)-Fe(1)	129.1(2)
C(1)-O(2)-Fe(1)#1	156.6(3)	C(2)-O(3)-Fe(1)#3	94.99(19)
C(6)-C(5)-C(2)	119.8(3)	O(5)-C(9)-C(10)	122.3(4)
N(1)-C(9)-C(10)	117.2(3)	C(21)-O(21)-Fe(1)	131.3(3)

4. Characterization of Metal−Organic Frameworks

Figure S5. PXRD patterns of Fe−NDI showing the calculated pattern based on the single-crystal (red) and as-synthesized Fe−NDI (blue).

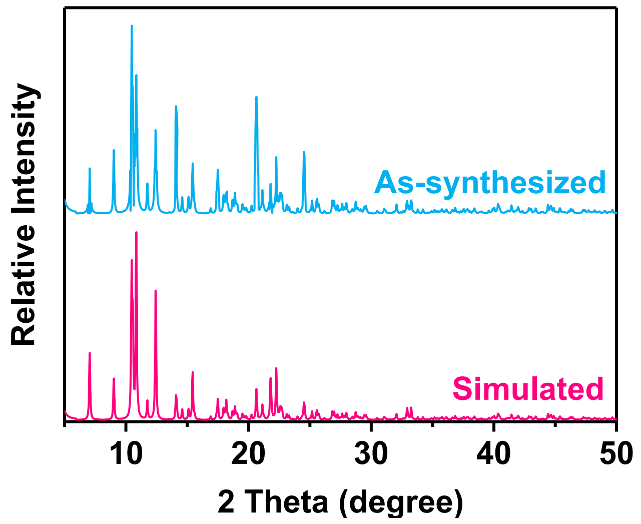

Figure S6. Energy-dispersive system elemental mapping images of Fe–NDI.

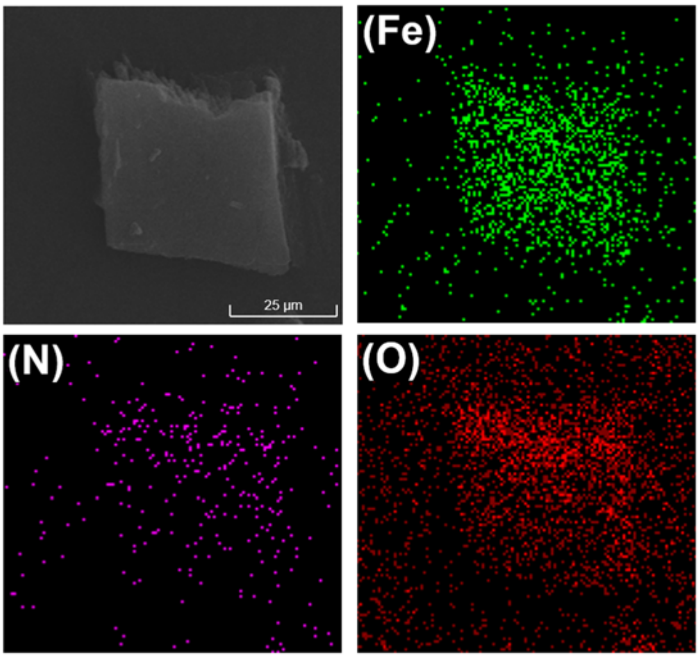

Figure S7. Thermogravimetric analyses (TGA) of Fe−NDI, exhibiting two steps of weight loss processes. The first weight loss event could be due to the loss of free and coordinated solvent. The second weight loss event was attributed to the decomposition of **Fe**−NDI.

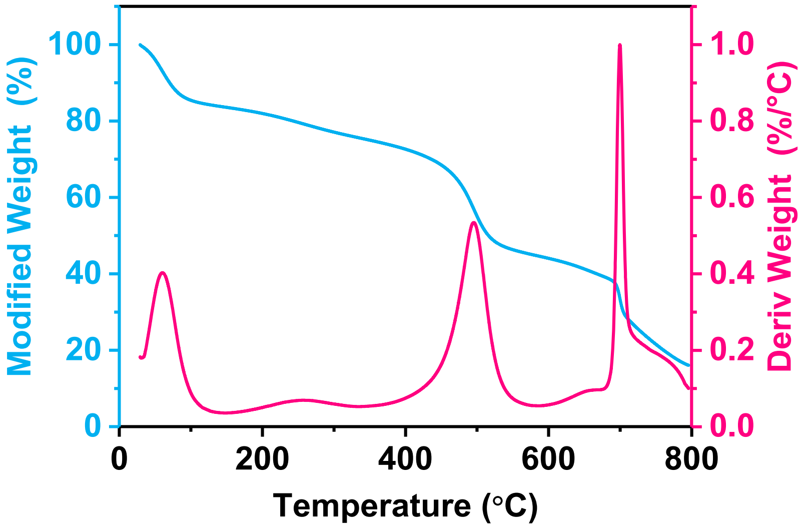

Figure S8. The IR spectra for Fe–NDI under different conditions. Fresh Fe–NDI (black line) was treated with CO_2 supercritical fluid extraction to obtain treated Fe–NDI (blue line) showed that the characteristic vibration peak of carbonyl group for DMF disappeared after treatment. Treated Fe–NDI that soaked in HCl (0.05 M) solution was recorded (red line) showed the stability of Fe–NDI.

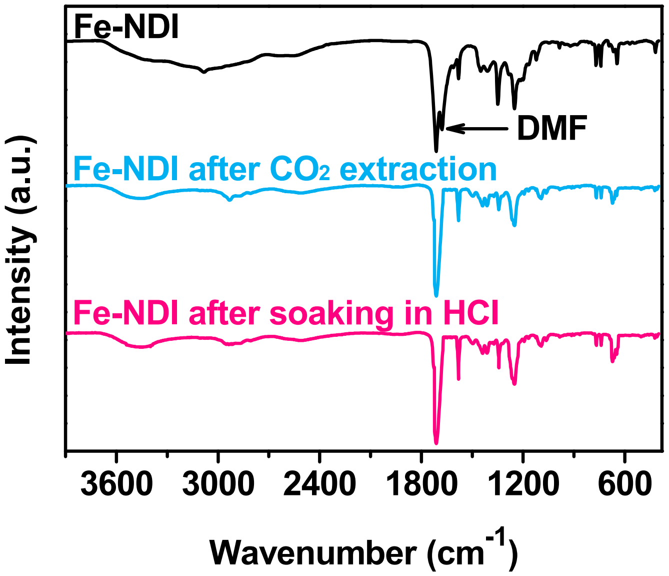

Figure S9. The stability test for Fe−NDI. Fe−NDI (20 mg) was soaked in different mixtures of aqueous hydrochloric acid (2 ml, pH=1.0−7.0) and acetonitrile (2 ml) for 12 h, respectively, then filtered, dried and weighted to calculate its recovery rate.

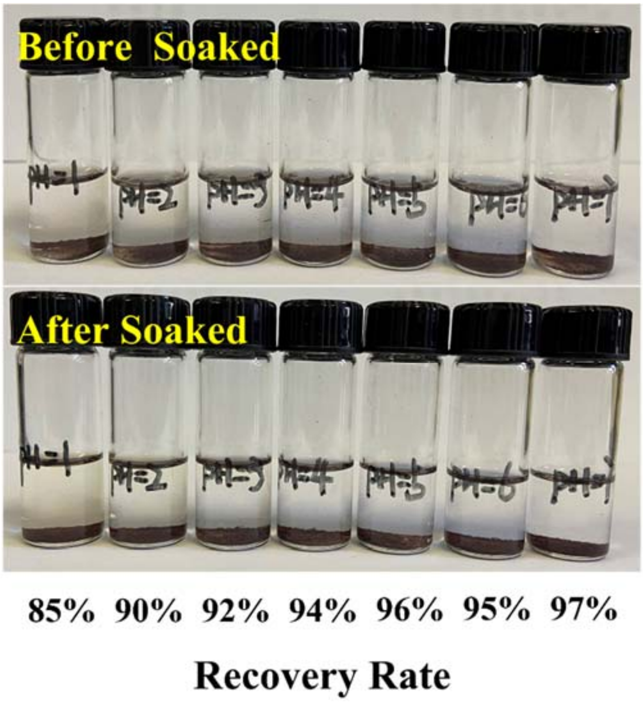

Figure S10. Solid UV-Vis spectrum of H_4BINDI (red line) and Fe−NDI (blue line).

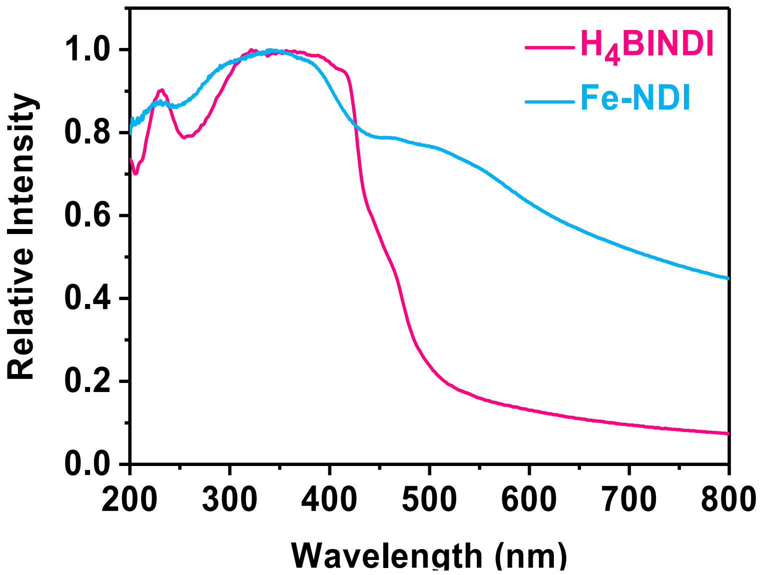

5. Data Relative to Photocatalytic Reaction

Figure S11. 455 nm LED bandwidth spectrum.

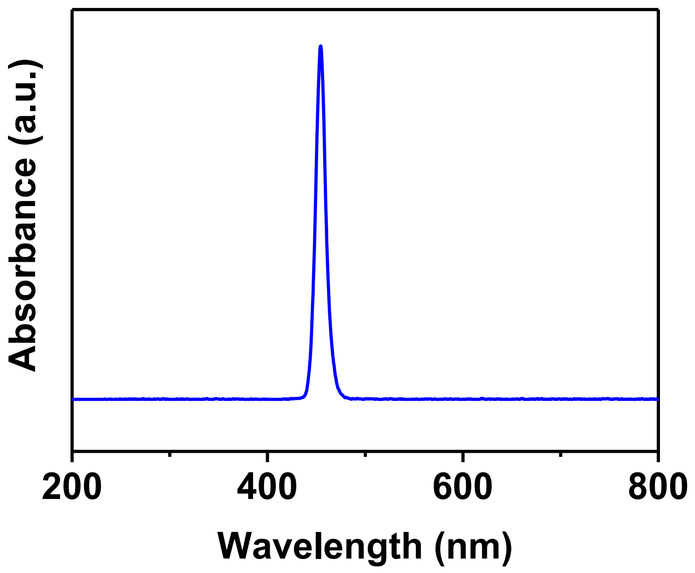

Figure S12. UV-Vis spectra of Fe–NDI (1 mg) in acetonitrile (3 mL) suspension before (black line) and after addition of HCl (12 M) (red line) and UV-Vis spectra of solution after removal of Fe–NDI (blue line).

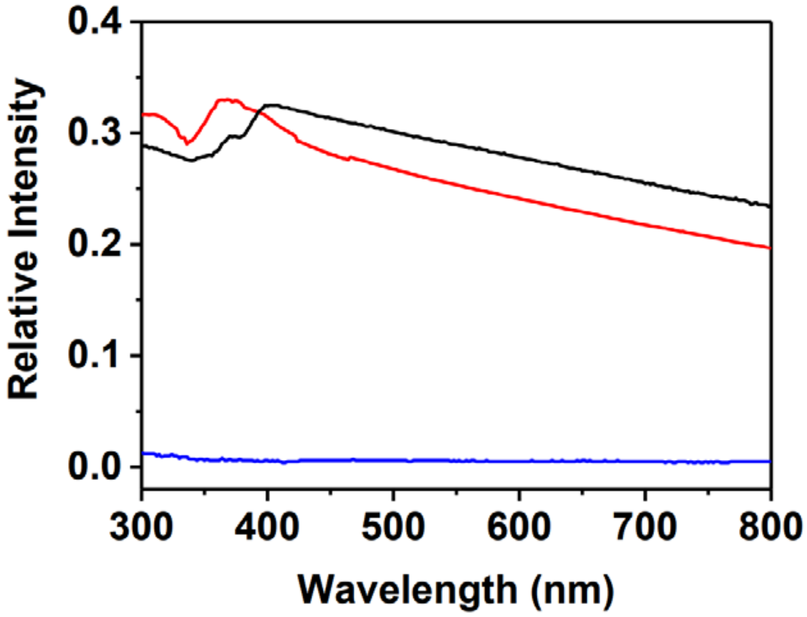

Table S4. The control experiments for C(sp^3)–H alkylation.

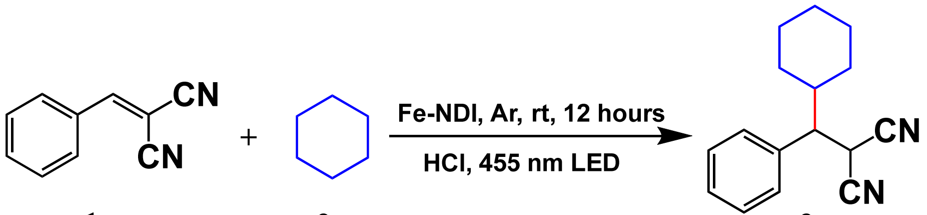

1a **2a** **3a**

Entry	Variation from standard conditions	Yield (%)
1	None	91
2	dark	N.R.
3	No Fe–NDI	N.R.
4	No HCl	N.R.

Standard conditions: Fe–NDI (5.0 μmol), **1a** (0.1 mmol), **2a** (1.0 mmol), HCl (0.05 mmol) and CH_3CN (1.0 mL) under 455 nm LED in Ar atmosphere at room temperature for 12 hours. Isolated yields were obtained by silica gel flash chromatography (EtOAc/petroleum ether). N.R. = No reaction.

Figure S13. The ESI-MS spectra of the Cl radical added products with styrene as the trapping agent.

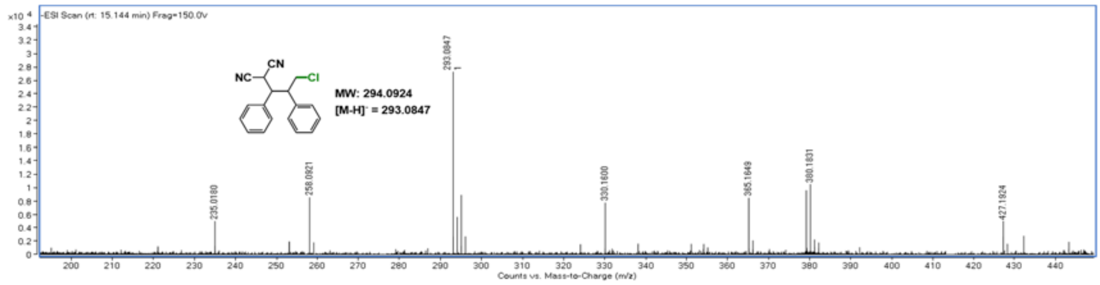

Figure S14. The ESI-MS spectra of the Cl radical with Ts protected bis-allyl amines added products $C_{13}H_{18}ClNO_2S$(a) and $C_{13}H_{17}Cl_2NO_2S$(b).

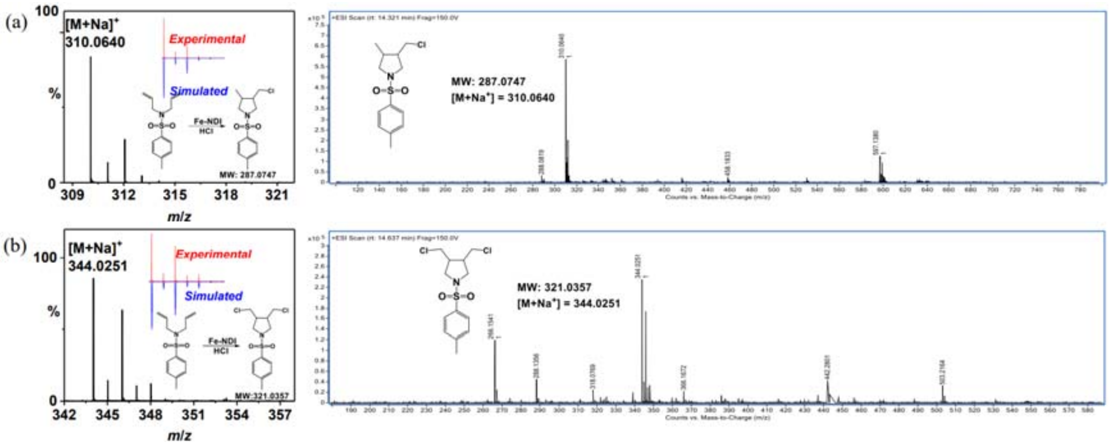

Figure S15. Reaction yields at different concentrations of HCl in C(sp^3)−H alkylation process. The reaction was performed with Fe−NDI (5.0 μmol), **1a** (0.1 mmol), **2a** (1.0 mmol), different amounts of HCl (12 M) and CH_3CN (1.0 mL) under 455 nm LED in Ar atmosphere at room temperature for 12 hours.

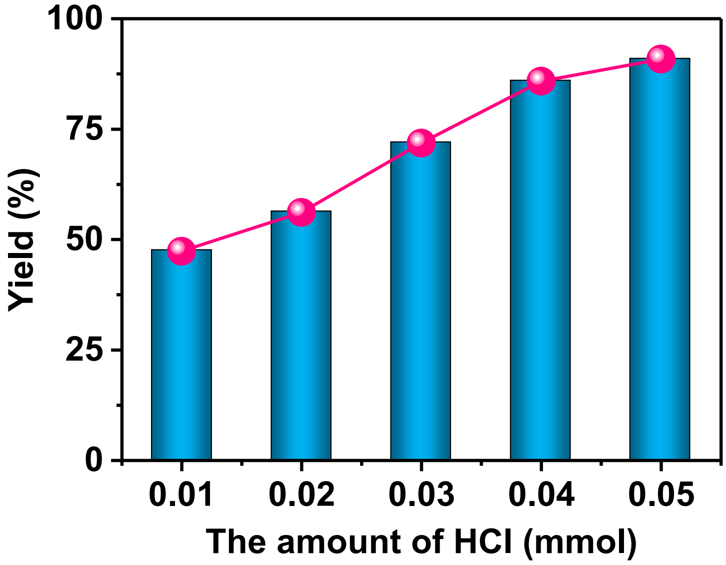

Figure S16. Time-dependent catalytic traces of C(sp^3)−H alkylation process. The reaction was performed with Fe−NDI (5.0 μmol), **1a** (0.1 mmol), **2a** (1.0 mmol), HCl (0.05 mmol 12 M) and CH_3CN (1.0 mL) under 455 nm LED in Ar atmosphere at room temperature.

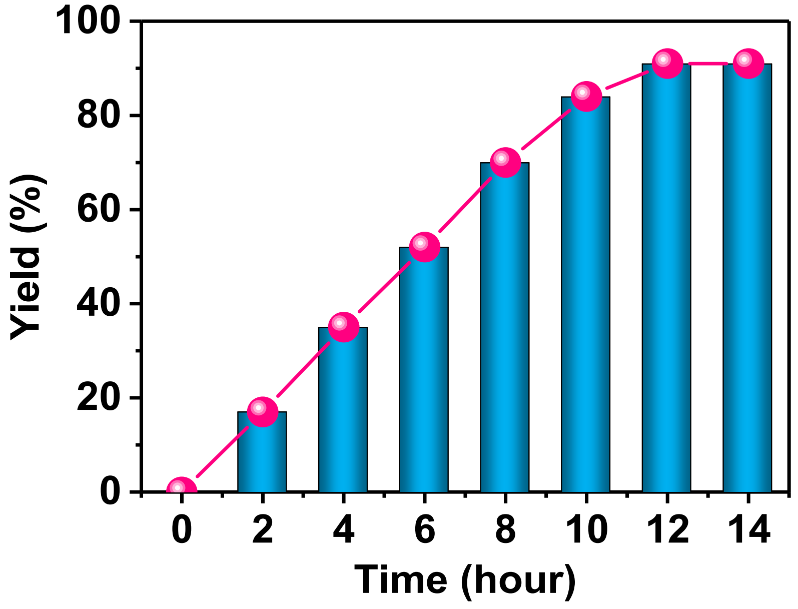

Figure S17. Time-dependent catalytic traces of C(sp^3)−H alkylation process conducted under standard conditions within 14 hours and with Fe−NDI filtrered after 4, 6 and 8 hours, respectively.

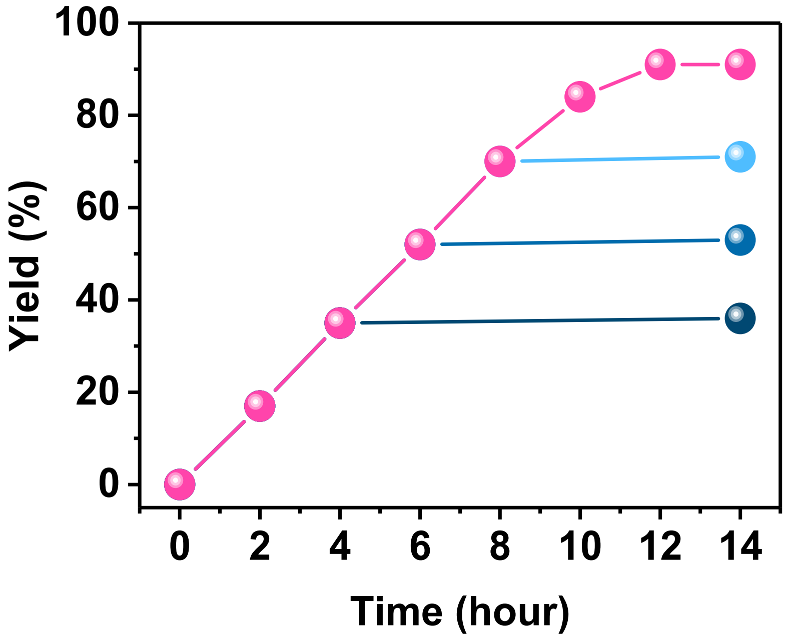

Figure S18. The scheme of the alkylation reaction.

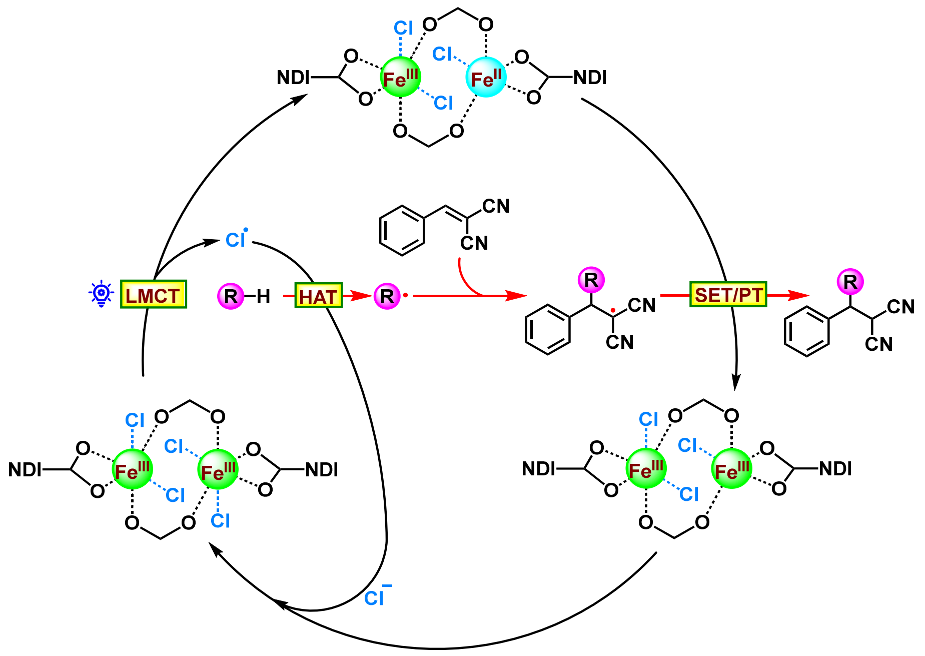

Figure S19. The cyclohexyl radical trapping experiment in direct C(sp^3)−H alkylation. The reaction was performed with Fe−NDI (5.0 μmol), benzylidene malononitrile (0.1 mmol), cyclohexane (1.0 mmol), TEMPO (0.3 mmol), HCl (0.05 mmol 12 M) and CH_3CN (1.0 mL) under 455 nm LED in Ar atmosphere at room temperature for 12 hours. Fe−NDI was filtered after finished reaction, and the reaction mixture was analyzed through ESI-MS. The ESI-MS spectra of TEMPO captured cyclohexane radicals.

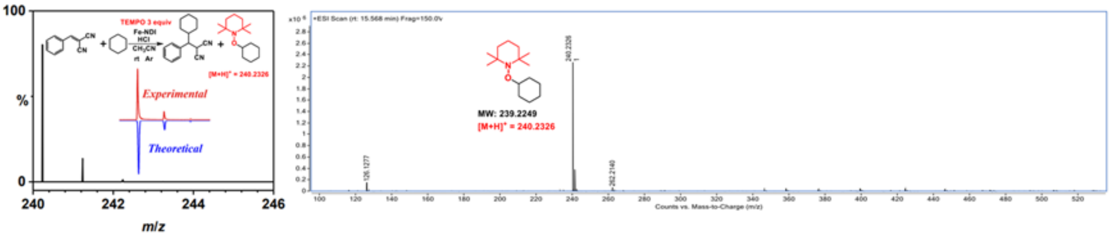

Figure S20. Fluorescence titration spectra of Fe–NDI (1 mg) in 1,4-dioxane (3 mL) suspension upon the addition of CHCA (1.5 mM).

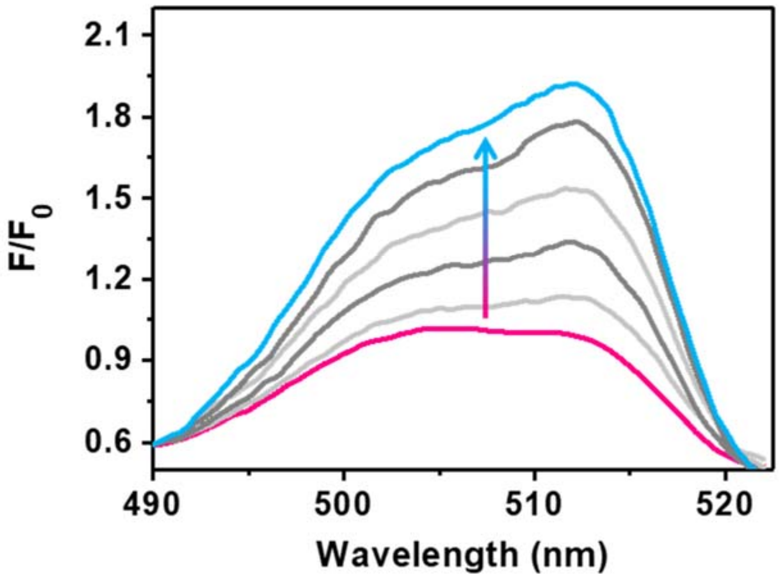

Figure S21. Fluorescence titration spectra of Fe–NDI (1 mg) in 1, 4-dioxane (3 mL) suspension before (black line) and after addition of CHCA (1.5 mM) (red line) and fluorescence spectra of solution after removal of Fe–NDI (blue line). There was a significant enhancement in the fluorescence emission of Fe−NDI after the addition of CHCA, while the fluorescence emission of the filtrate became disappear after separating Fe−NDI from the system by filtration. This result suggested that the fluorescence enhancement was due to the interaction of CHCA with heterogeneous Fe−NDI and excluded the dissociation of Fe−NDI.

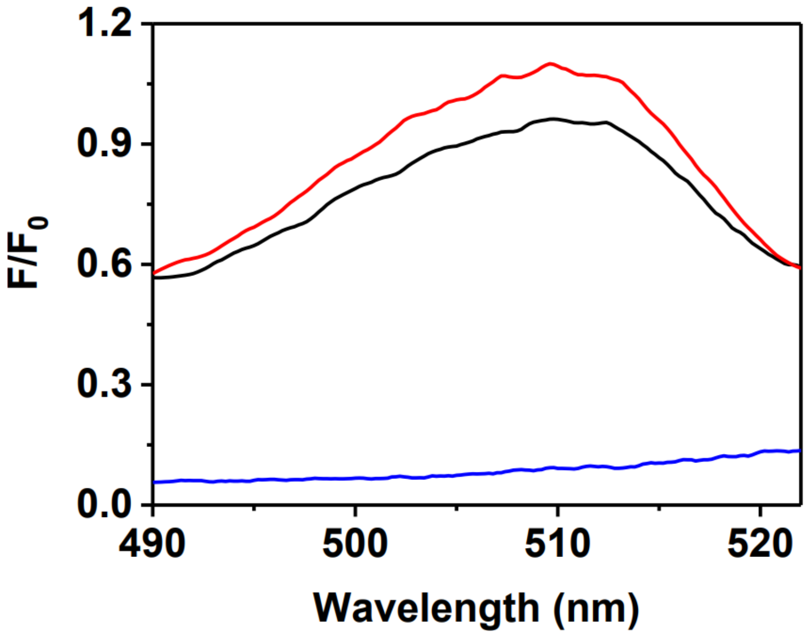

Figure S22. The ESI-MS spectra of cyclohexyl radical capture during decarboxylation functionalisation using 2,2,6,6- tetramethylpiperidinooxy (TEMPO) as a trapping agent.

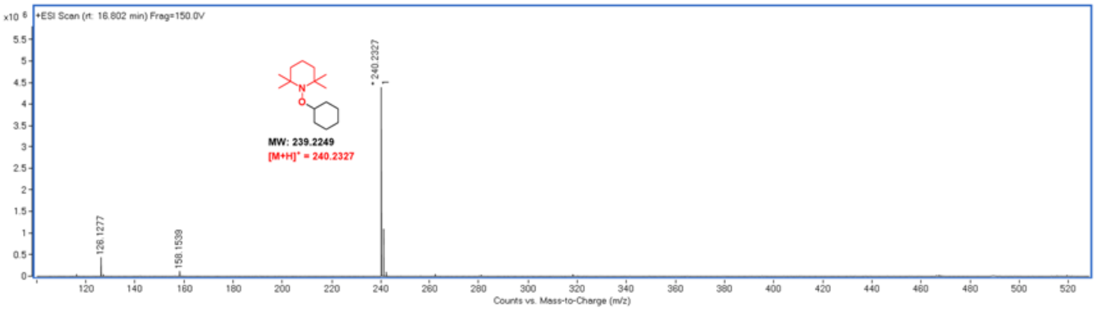

Figure S23. The scheme of the decarboxylation reaction.

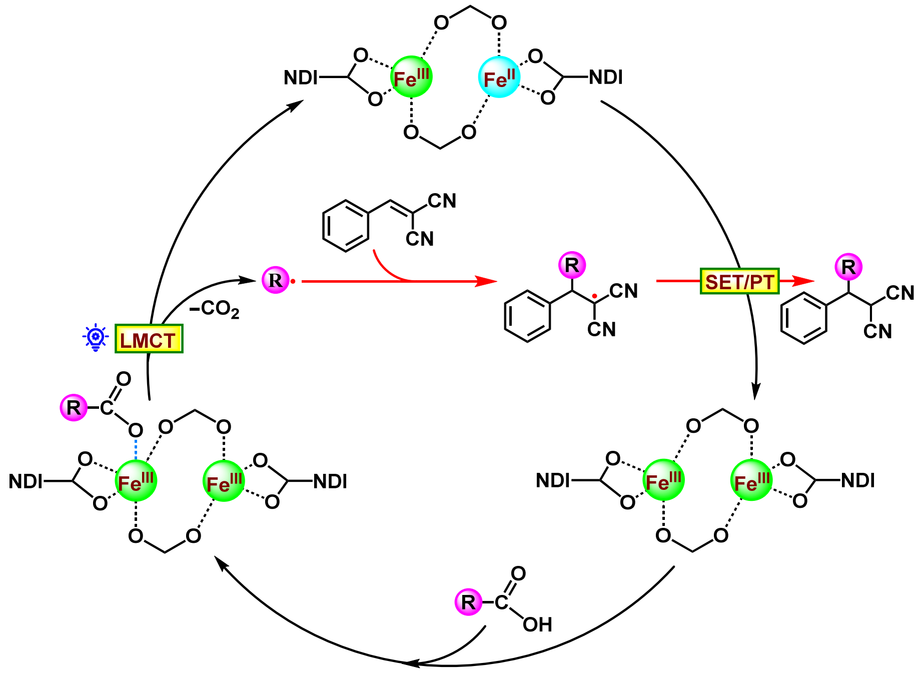

Figure S24. Raman spectrum of Fe−NDI during the oxidation reaction.

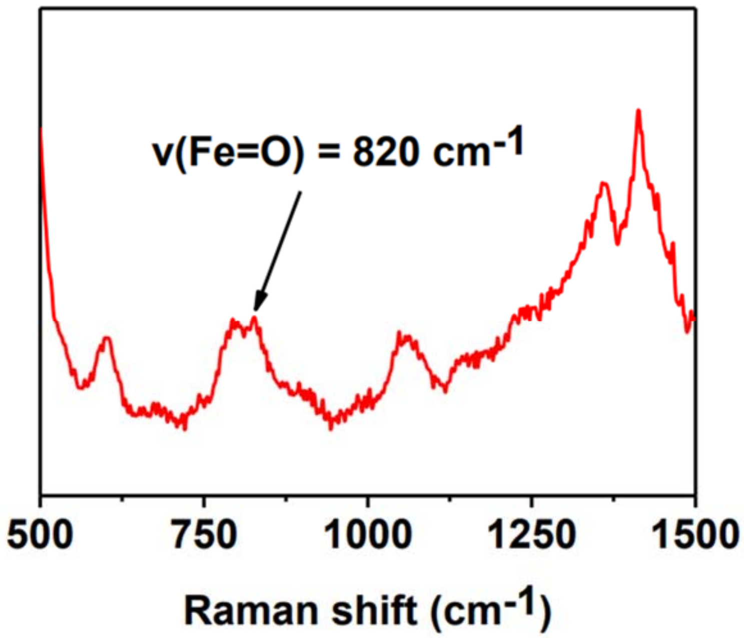

6. DFT Calculations

We have employed the Vienna Ab initio Simulation Package (VASP) to perform all density functional theory (DFT) calculations within the generalized gradient approximation (GGA) using the Perdew-Burke-Ernzerhof (PBE) unctional. We have chosen the projected augmented wave (PAW) potentials to describe the ionic cores and take valence electrons into account using a plane wave basis set with a kinetic energy cutoff of 450 eV. Spin-polarization effect was also considered. Geometry optimizations were performed with the force convergency smaller than 0.05 eV/Å. The DFT-D3 empirical correction method was employed to describe van der Waals interactions. Monkhorst-Pack k-points of 2×2×3 was applied for all the calculations. All atoms are relaxed in all the calculations.

Table S5. Gibbs free energy calculations of each step of the intermediate for the oxidation processes. S for steady state, TS for transition state and T for terminal state .

	Pathway	ΔG (eV)
S1-S2	$O_2 \rightarrow O_2^*$	-0.678
S2-S3	$O_2^* + H^+ + e^- \rightarrow OOH^*$	-0.384
S3-TS1	$OOH^* \rightarrow Fe\cdots OO(H){-}Fe^*$	+0.941
TS1-S4	$Fe\cdots OO(H){-}Fe^* + H^+ \rightarrow O^* + H_2O$	-1.776
S4-S5	$O^* + C_6H_{11}^\bullet \rightarrow C_6H_{11}O^*$	-2.188
S5-S6	$C_6H_{11}O^* \rightarrow C_6H_{10}O^* + H^+$	-0.560
S6-T1	$C_6H_{10}O^* + Cl^- \rightarrow C_6H_{10}O + Cl^* + e^-$	-0.287

Figure S25. The proposed reaction intermediates of the C–H activation and oxidation pathway for cyclohexane on the Fe−NDI.

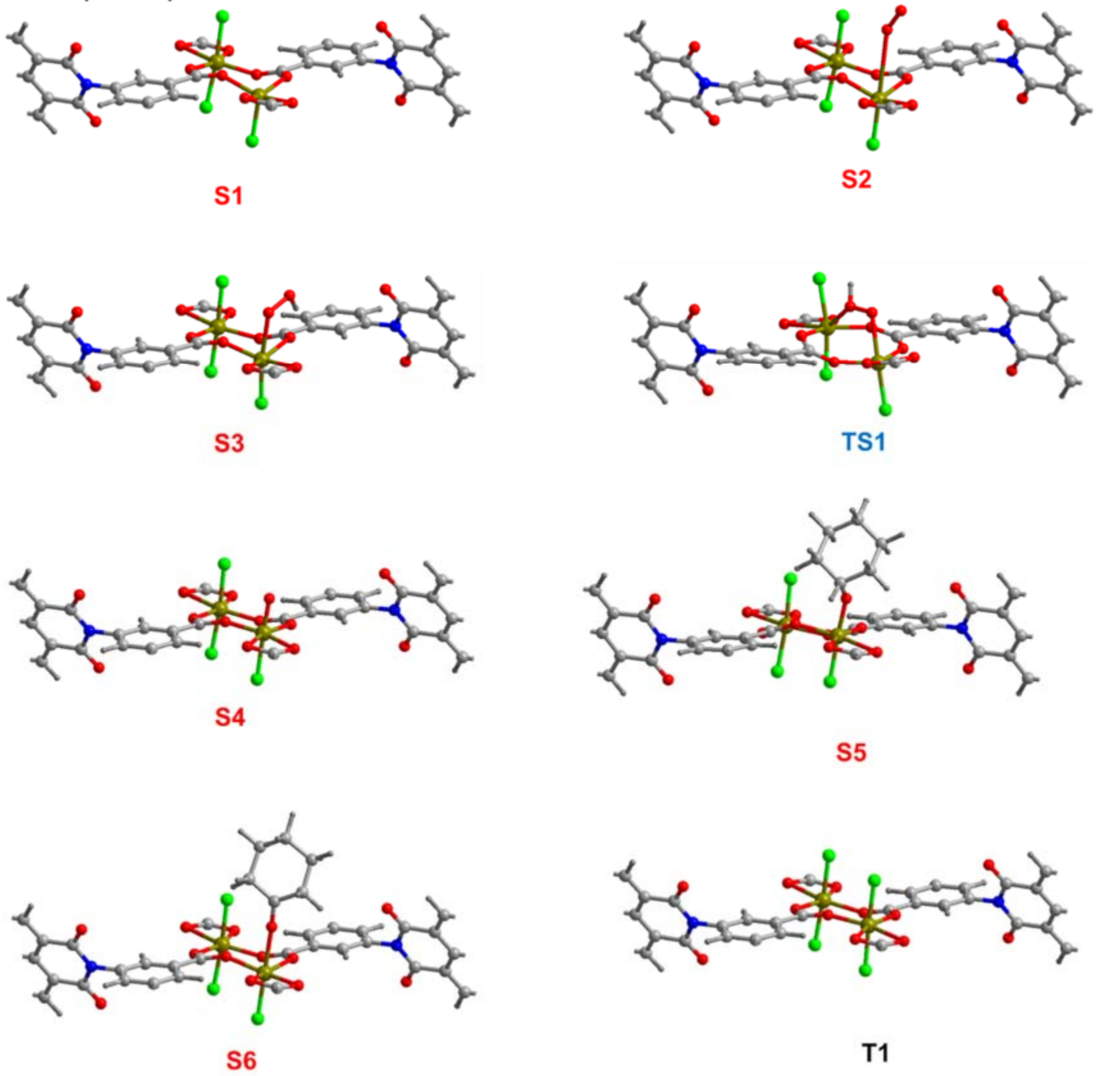

7. The ^{1}H / ^{13}C NMR Spectra of C(sp^3)−H alkylation products

3a: 2−(2−Cyclohexyl−1−phenylethyl)malononitrile

White oil. Eluent: CH_2Cl_2. ^{1}H NMR (400 MHz, $CDCl_3$) δ 7.42–7.30 (m, 5H), 4.20–4.18 (d, J = 8.0 Hz, 1H), 2.90–2.86 (m, 1H), 2.05–1.82 (m, 3H), 1.69–1.64 (m, 2H), 1.48–1.35 (m, 2H), 1.21−1.04 (m, 3H), 0.86−0.77 (m, 1H); ^{13}C NMR (101 MHz, $CDCl_3$) δ 136.73, 129.13, 128.73, 128.32, 112.28, 112.00, 52.31, 39.23, 31.18, 30.58, 27.13, 25.82, 25.74.

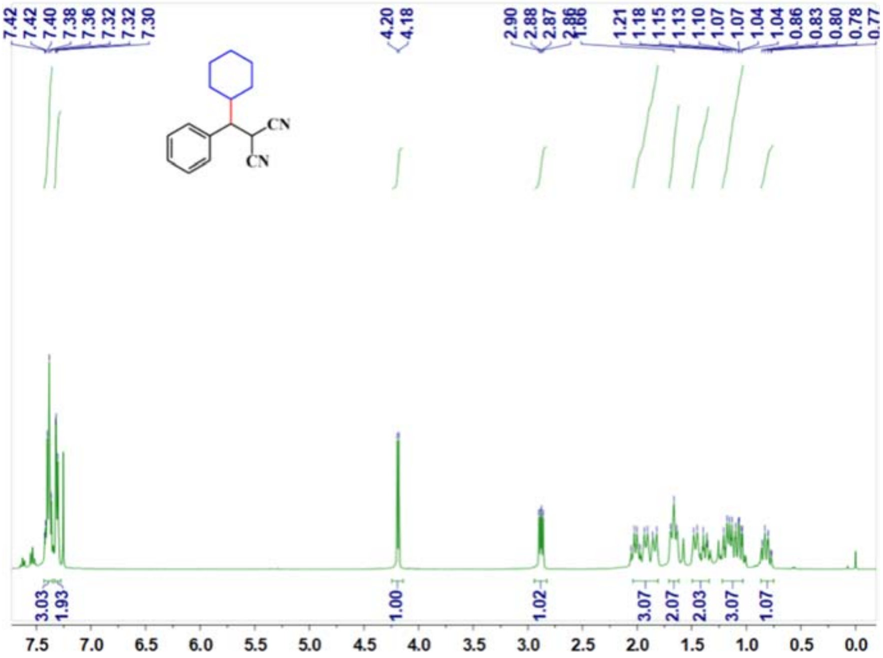

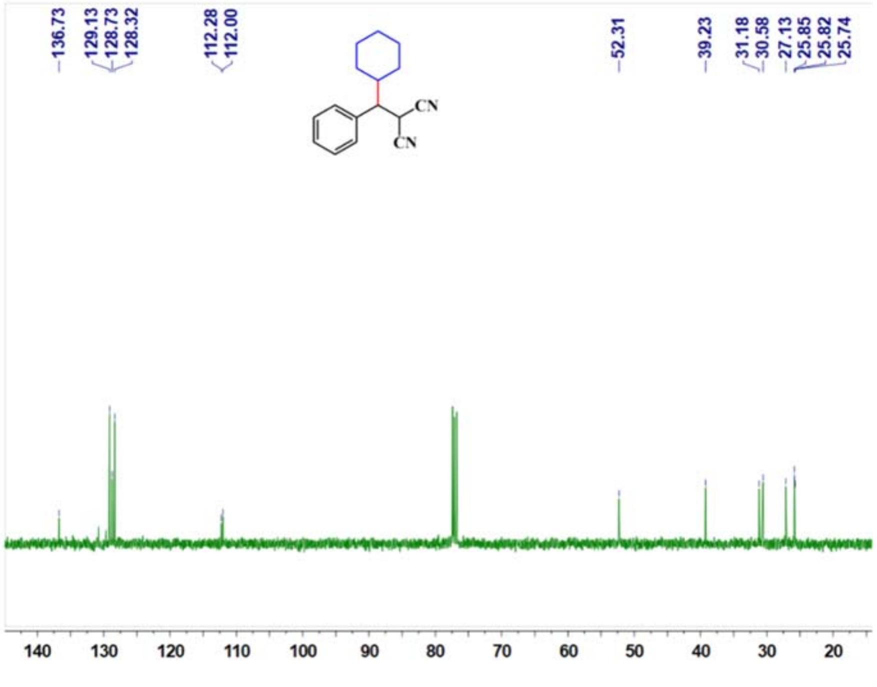

3b: 2−(2−Cyclopentyl−1−phenylethyl)malononitrile

White oil. Eluent: CH_2Cl_2. ^{1}H NMR (400 MHz, $CDCl_3$) δ 7.41–7.36 (m, 5H), 4.08–4.07 (d, J = 4.0 Hz, 1H), 2.94–2.91 (m, 1H), 2.59–2.53 (m, 1H), 2.07−2.04 (m, 1H), 1.79–1.70 (m, 2H), 1.59−1.57 (m, 2H), 1.30−1.25 (m, 2H), 1.07−1.03 (m, 1H); ^{13}C NMR (101 MHz, $CDCl_3$) δ 137.34, 129.08, 128.77, 128.19, 112.04, 111.80, 52.31, 42.40, 31.67, 31.63, 29.16, 25.47, 24.75.

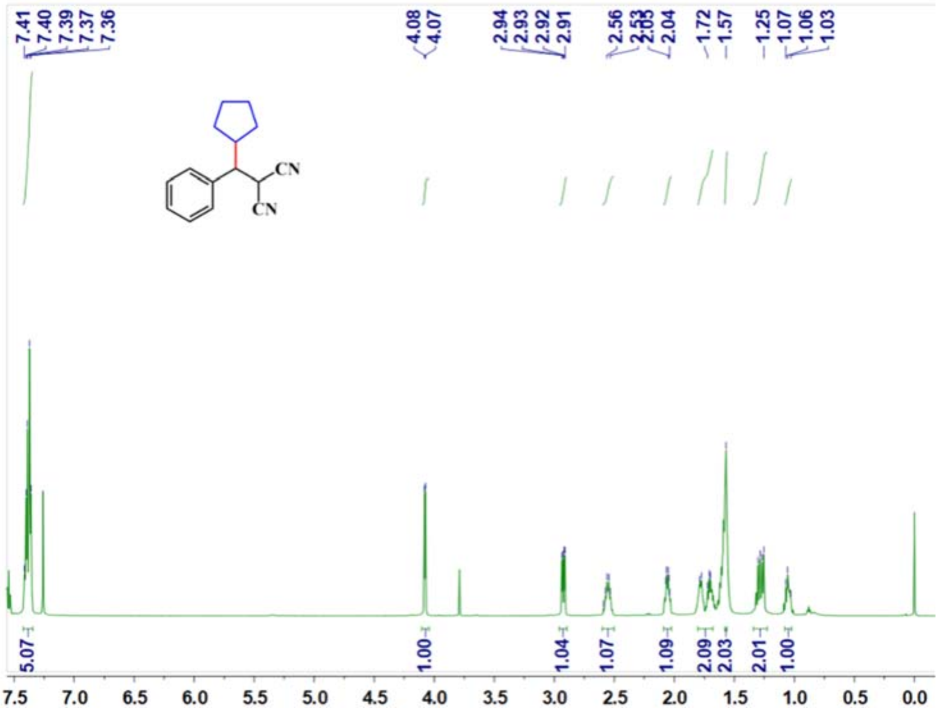

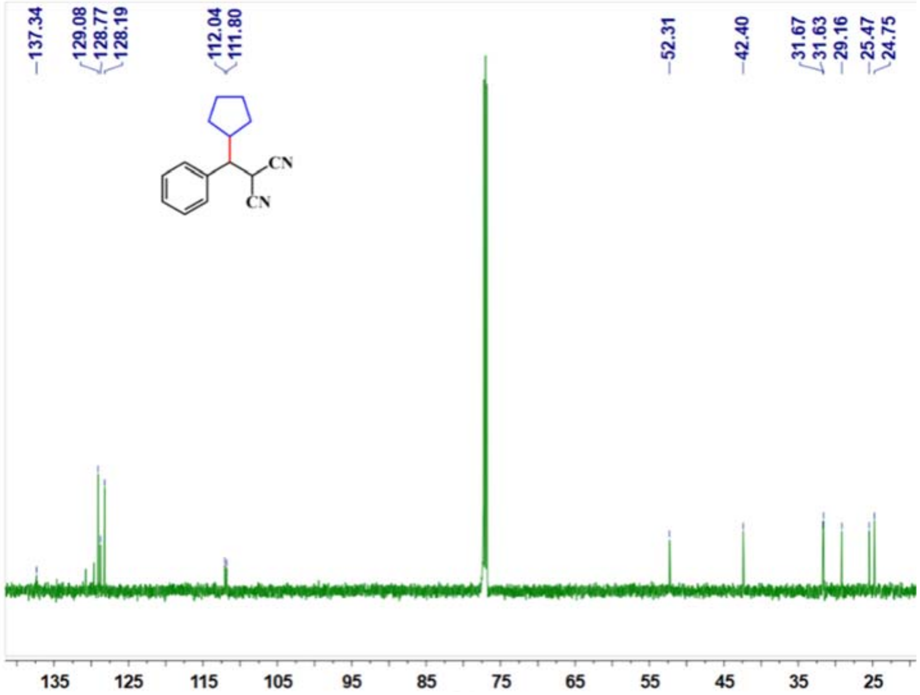

3c: 2-(cyclooctyl(phenyl)methyl)malononitrile

White oil. Eluent: CH_2Cl_2. ^1H NMR (400 MHz, $CDCl_3$) δ 7.43–7.36 (m, 5H), 4.23–4.21 (d, J = 8.0 Hz, 1H), 3.00–2.96 (m, 1H), 2.37–2.35 (m, 1H), 1.88−1.78 (m, 2H), 1.65–1.47 (m, 10H), 1.31−1.24 (m, 2H); ^{13}C NMR (101 MHz, $CDCl_3$) δ 136.97, 129.11, 128.75, 128.48, 112.25, 111.88, 52.14, 38.17, 30.59, 28.62, 27.41, 26.62, 26.33, 25.74, 24.31.

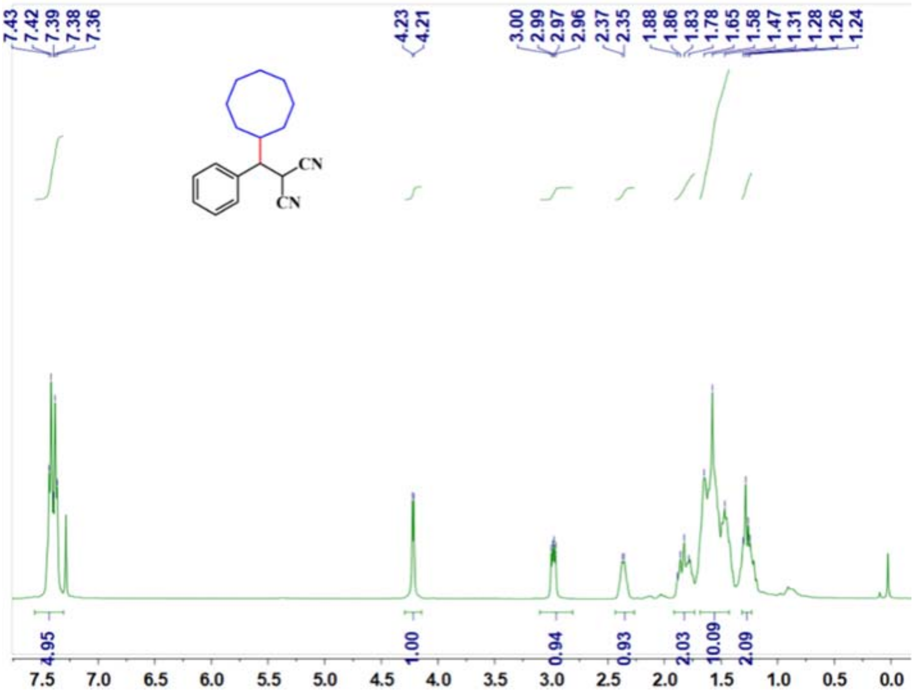

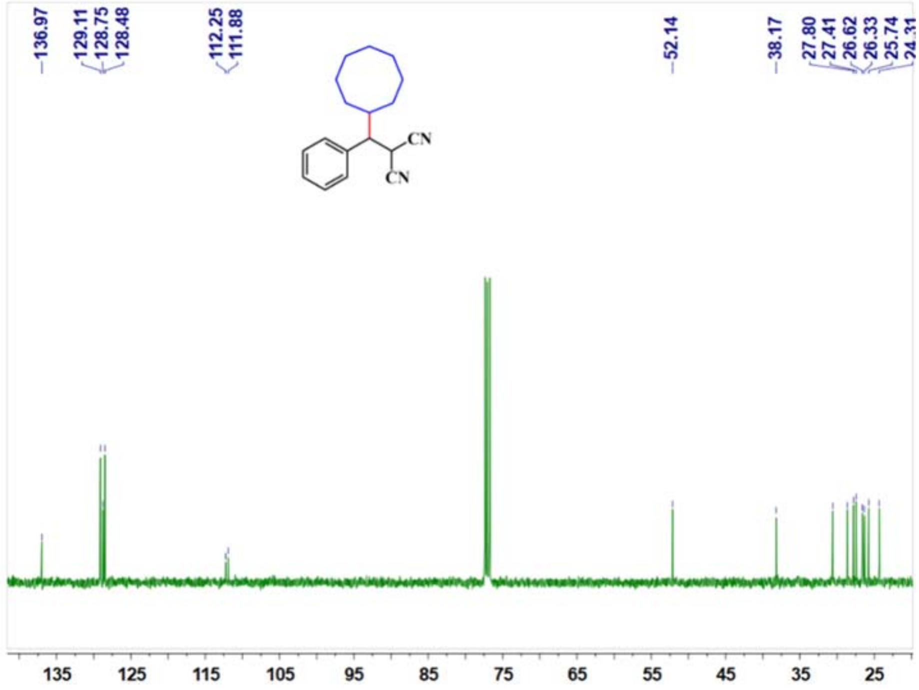

3d: 2−[2−(2,5−Dioxyl)−1−phenylethyl]malononitrile

White oil. Eluent: CH_2Cl_2. ^{1}H NMR (400 MHz, $CDCl_3$) δ 7.44–7.37 (m, 5H), 4.57–4.56 (d, J = 4.0 Hz, 1H), 4.13–4.07 (m, 1H), 3.92–3.81 (m, 2H), 3.77–3.74 (m, 1H), 3.61–3.55 (m, 1H), 3.52−3.49 (m, 1H), 3.23−3.17 (m, 1H), 3.13−3.09 (m, 1H); ^{13}C NMR (101 MHz, $CDCl_3$) δ 132.56, 129.76, 129.59, 128.38, 111.98, 111.50, 73.82, 69.32, 67.19, 66.22, 48.47, 26.08.

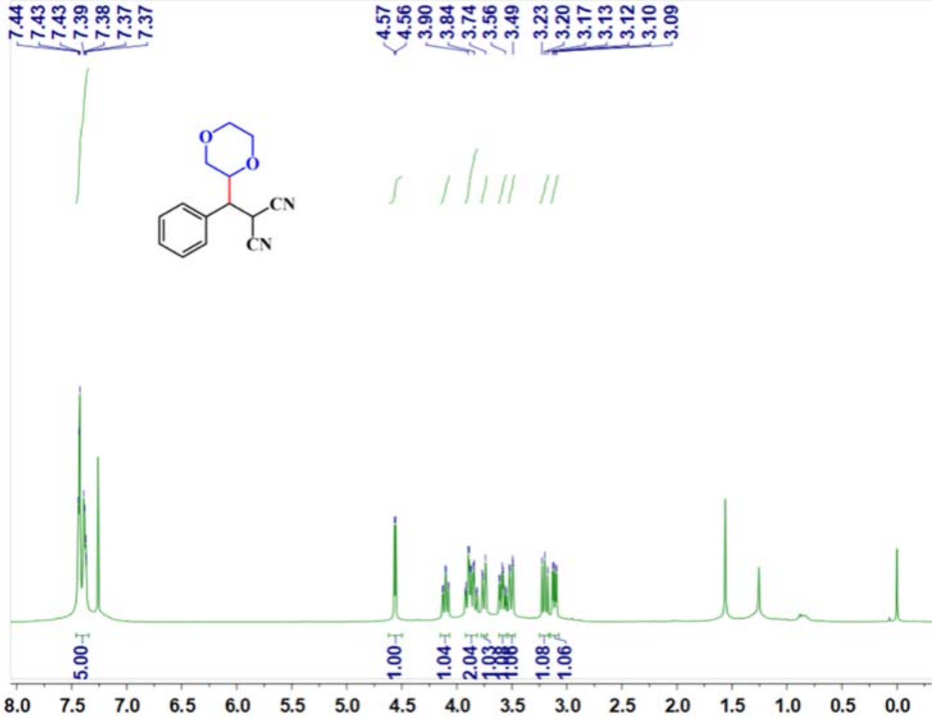

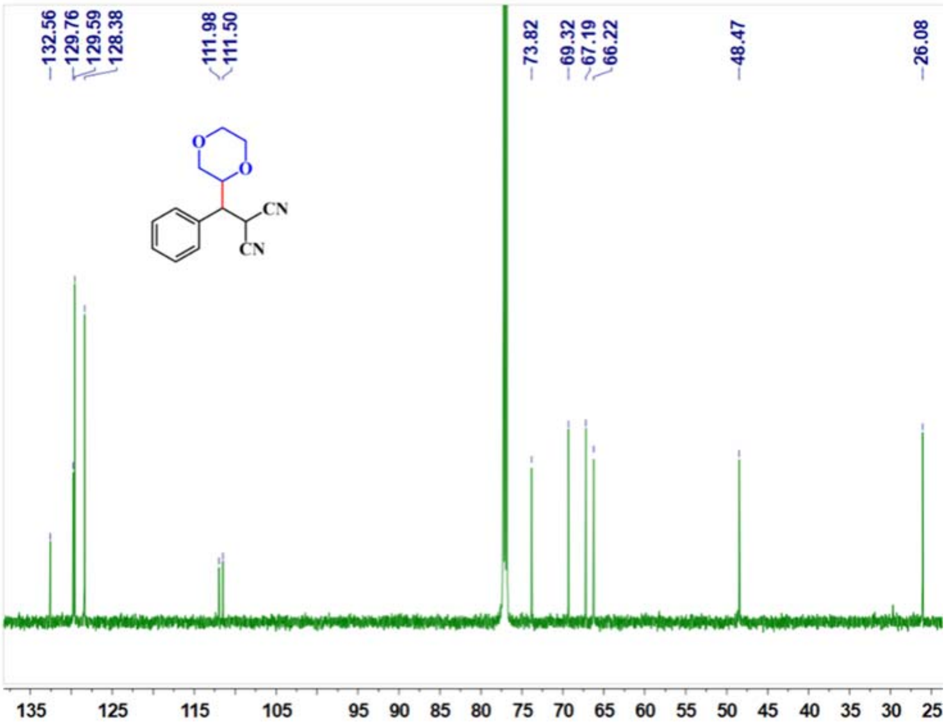

3e: 2-((1,3-dioxolan-2-yl)(phenyl)methyl)malononitrile

White oil. Eluent: CH_2Cl_2. ^1H NMR (400 MHz, $CDCl_3$) δ 7.47–7.44 (m, 5H), 5.28–5.27 (d, J = 4.0 Hz, 1H), 4.33–4.31 (d, J = 8.0 Hz, 1H), 4.16–4.05 (m, 2H), 4.02–3.93 (m, 2H), 3.56–3.53 (m, 1H); ^{13}C NMR (101 MHz, $CDCl_3$) δ 133.14, 129.34, 129.24, 128.96, 112.00, 111.77, 102.92, 65.94, 65.06, 49.33, 24.30.

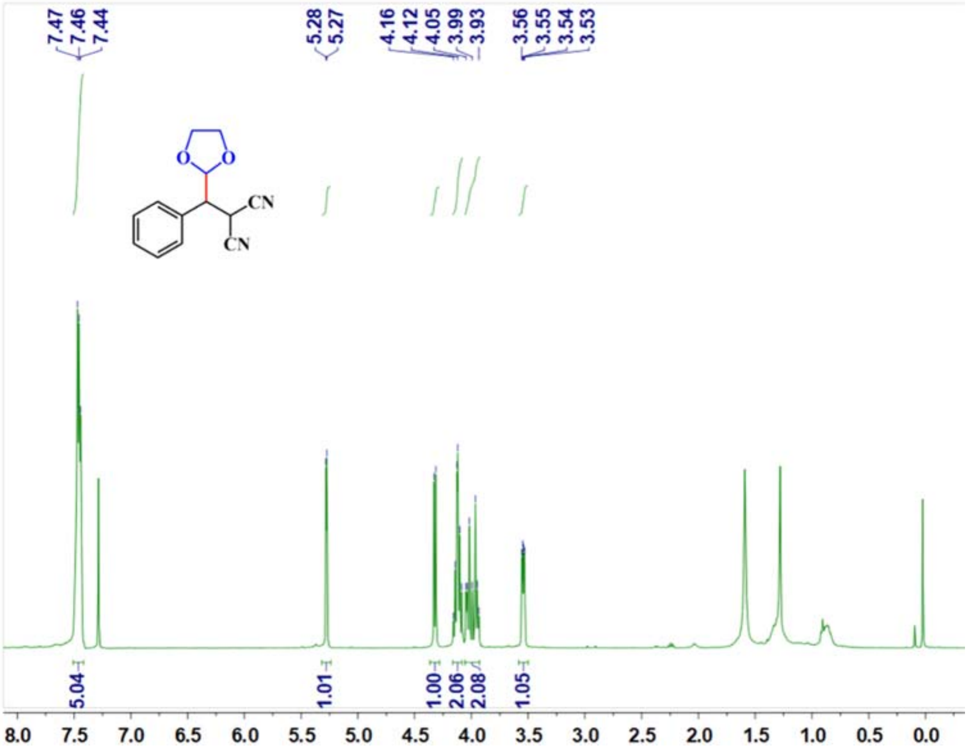

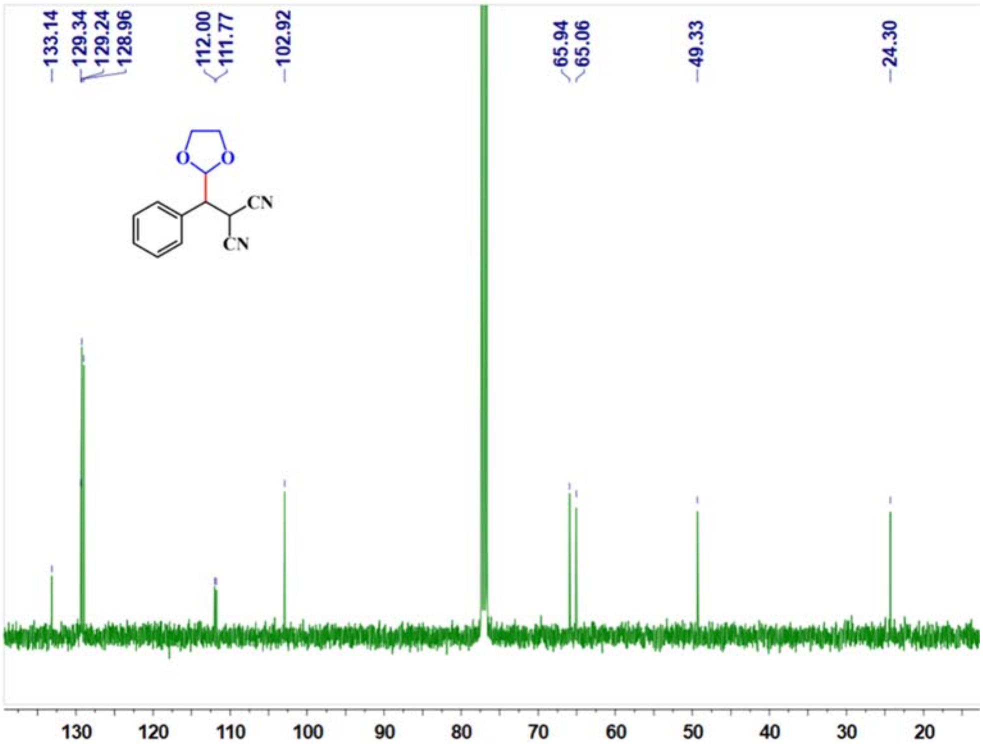

3f: 2−[2−(Tetrahydro−2H−pyran−4−yl)−1−phenylethyl]malononitrile

White oil. Eluent: CH_2Cl_2. ^1H NMR (400 MHz, $CDCl_3$) δ 7.43–7.39 (m, 3H), 7.37–7.36 (m, 2H), 4.62−4.61 (d, J = 4.0 Hz, 1H), 4.05–3.99 (m, 2H), 3.75−3.71 (m, 1H), 3.13−3.11 (m, 1H), 1.83−1.74 (m, 2H), 1.65−1.63 (m, 2H), 1.43−1.37 (m, 2H); ^{13}C NMR (101 MHz, $CDCl_3$) δ 135.11, 129.37, 129.15, 128.53, 112.52, 112.22, 70.12, 52.43, 33.54, 30.56, 26.69. 26.26, 25.67.

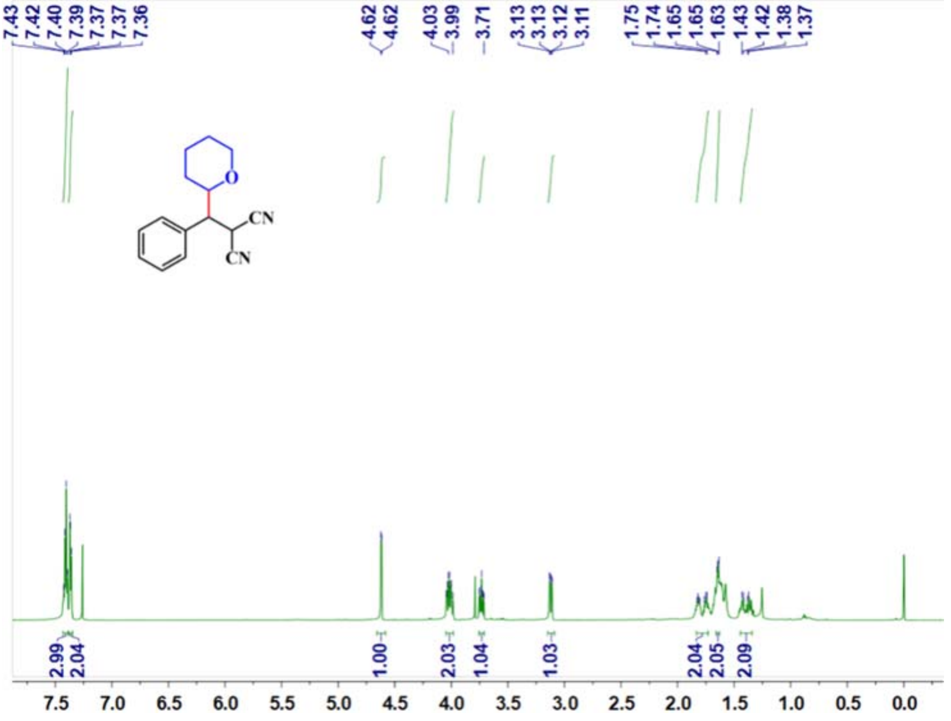

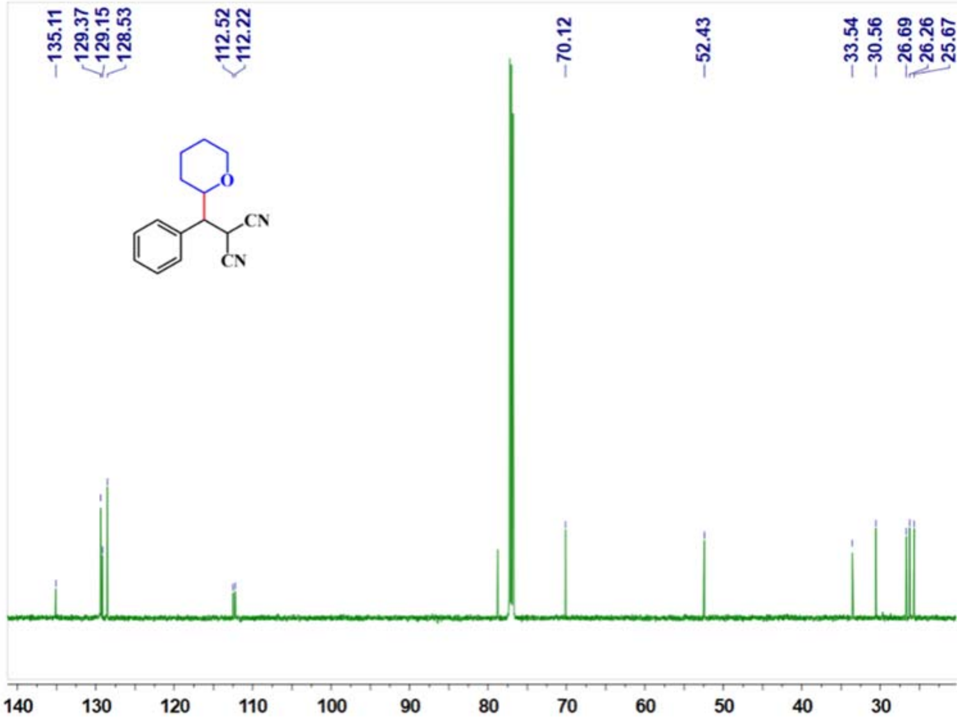

3g: 2−[2−(1−Carbonyl−1−methyl)−1−phenylethyl]malononitrile

White oil. Eluent: CH_2Cl_2. ^1H NMR (400 MHz, $CDCl_3$) δ 7.45–7.43 (m, 3H), 7.38–7.36 (m, 2H), 5.42−5.37 (m, 1H), 4.22−4.21 (d, J = 4.0 Hz, 1H), 3.34−3.31 (m, 1H), 2.18 (s, 3H), 1.17−1.16 (d, J = 4.0 Hz, 3H); ^{13}C NMR (101 MHz, $CDCl_3$) δ 169.84, 134.14, 129.67, 128.30, 111.65, 111.40, 70.79, 51.44, 26.94, 21.14, 18.75.

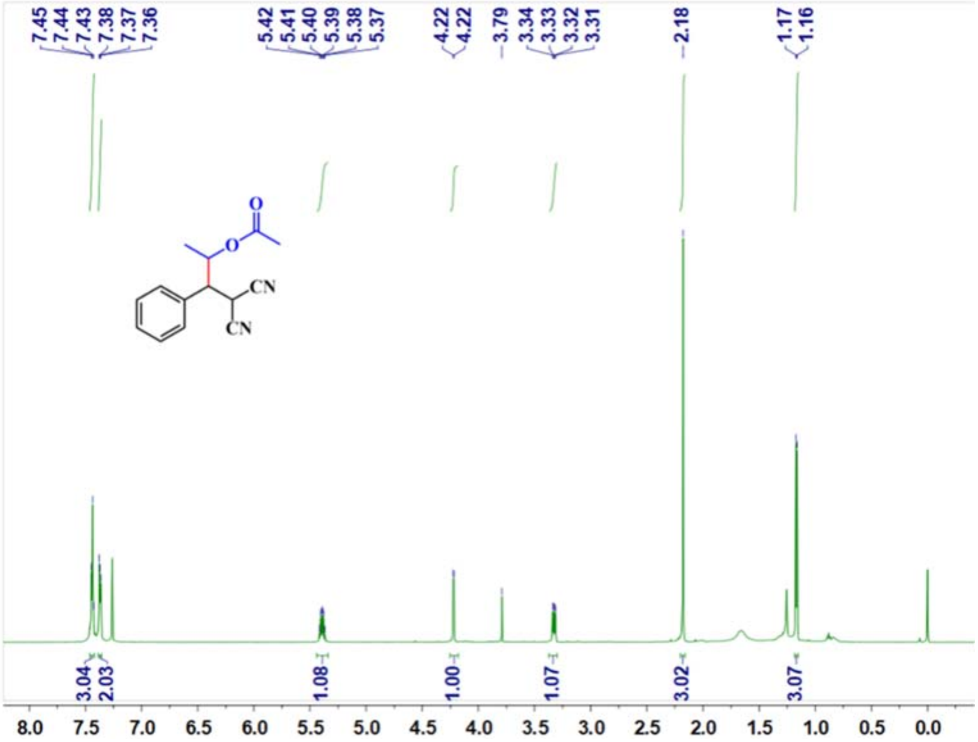

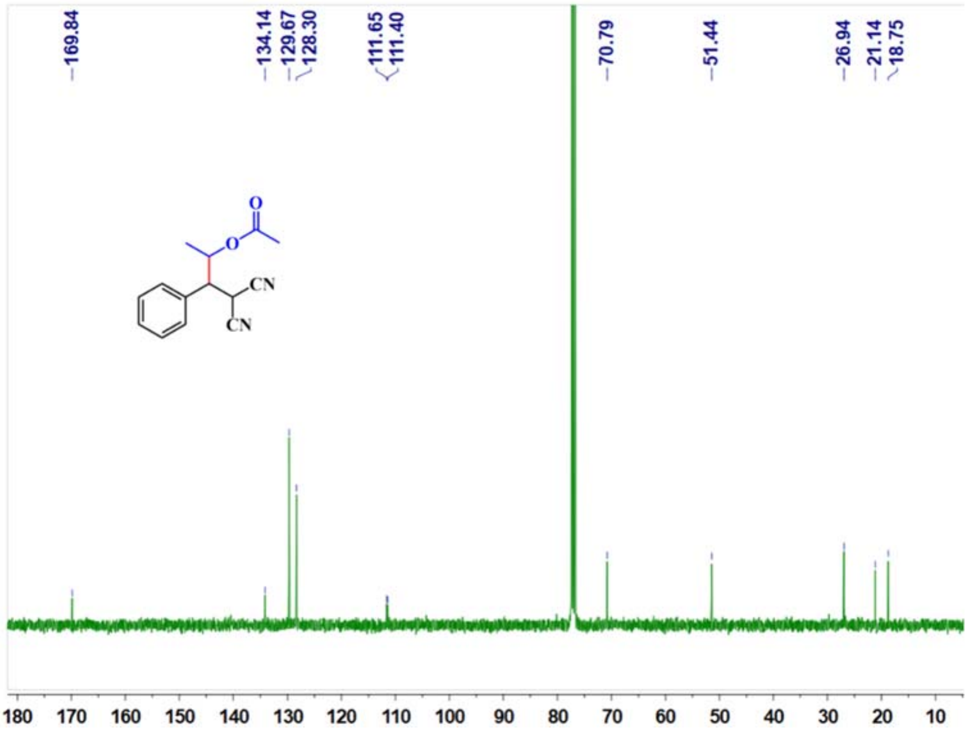

3h: 2−(2−Benzyl−1−phenylethyl)malononitrile

White oil. Eluent: CH_2Cl_2. ^{1}H NMR (400 MHz, $CDCl_3$) δ 7.45–7.41 (m, 5H), 7.38–7.32 (m, 3H), 7.32−7.21 (m, 2H), 3.89–3.87 (d, J = 8.0 Hz, 1H), 3.51−3.46 (m, 1H), 3.31−3.29 (m, 2H); ^{13}C NMR (101 MHz, $CDCl_3$) δ 136.64, 136.42, 129.22, 129.18, 129.10, 128.93, 128.04, 127.61, 112.07, 111.45, 48.35, 38.52, 28.53.

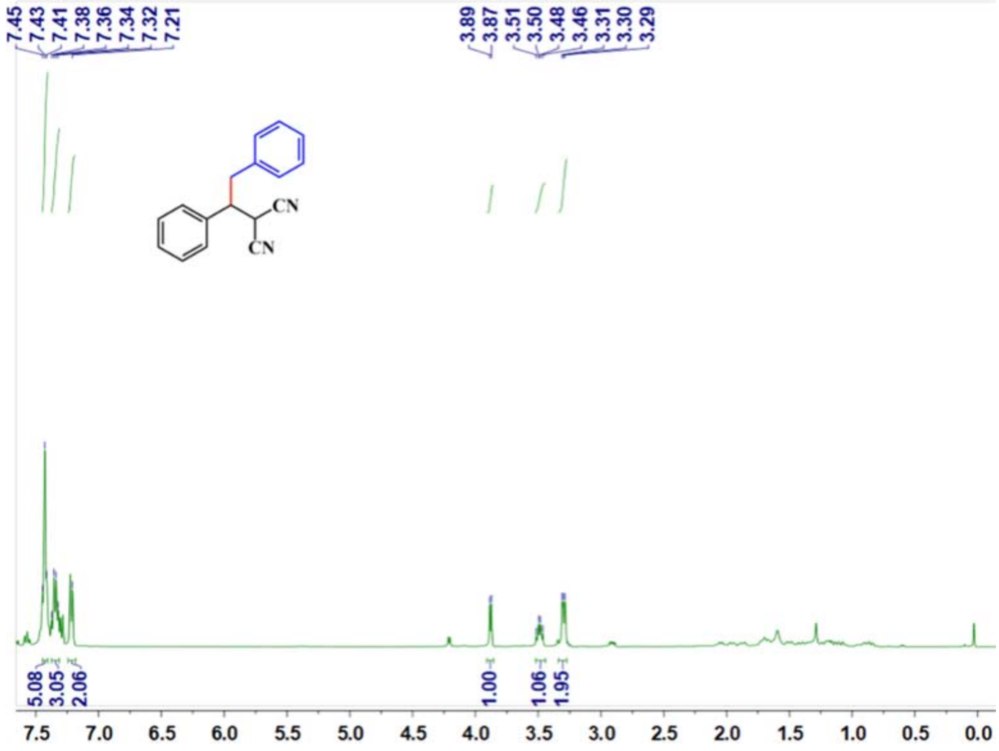

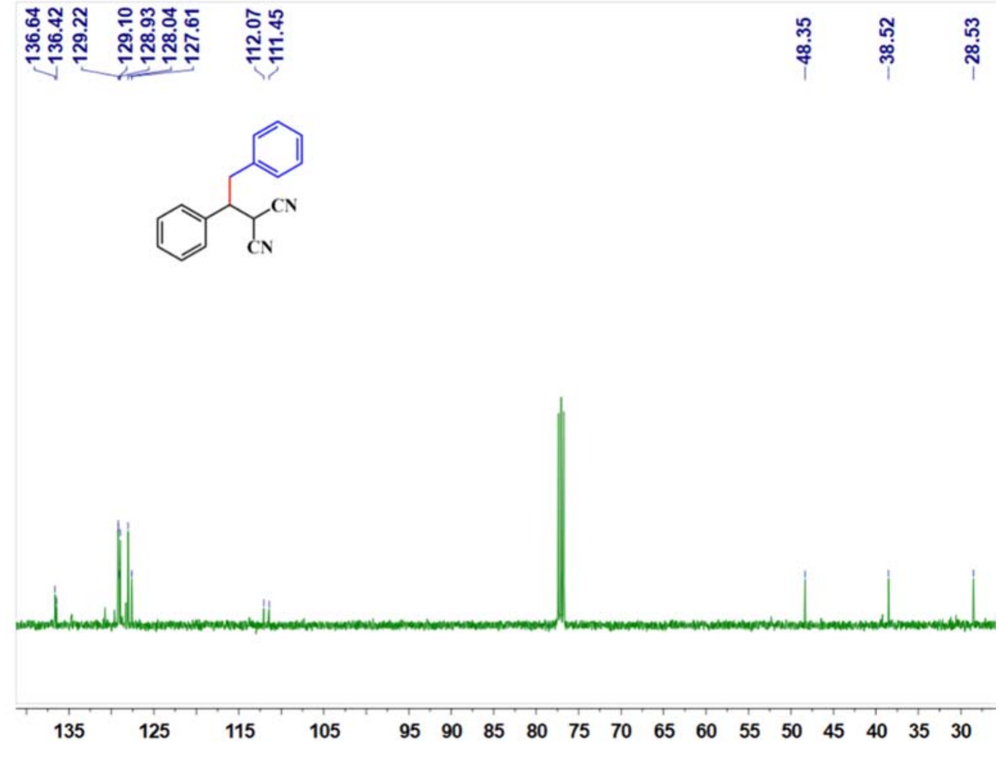

3i: 2−(2−Methoxy−1−phenylethyl)malononitrile

White oil. Eluent: CH_2Cl_2. ^{1}H NMR (400 MHz, $CDCl_3$) δ 7.39 – 7.22 (m, 5H), 3.77 (d, J = 6.1 Hz, 1H), 3.38 (p, J = 6.5 Hz, 1H), 1.58 (d, J = 7.0 Hz, 3H); ^{13}C NMR (101 MHz, $CDCl_3$) δ138.22, 129.27, 128.87, 127.23, 111.95, 111.66, 41.25, 31.22, 17.79.

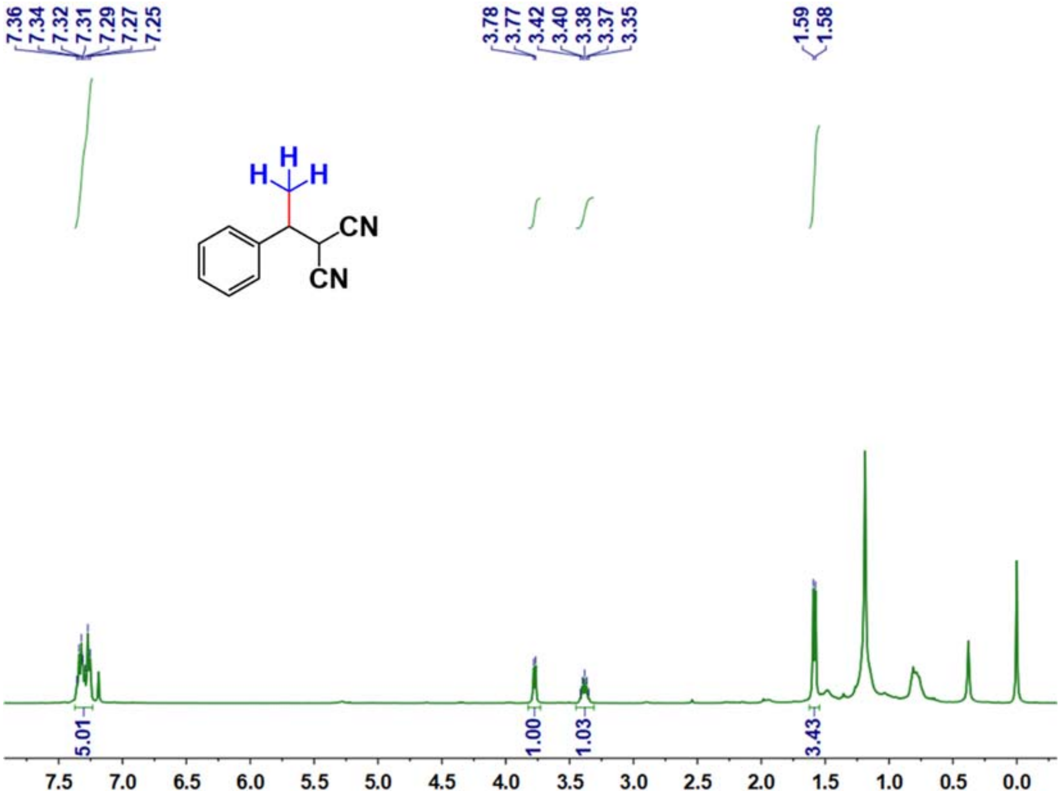

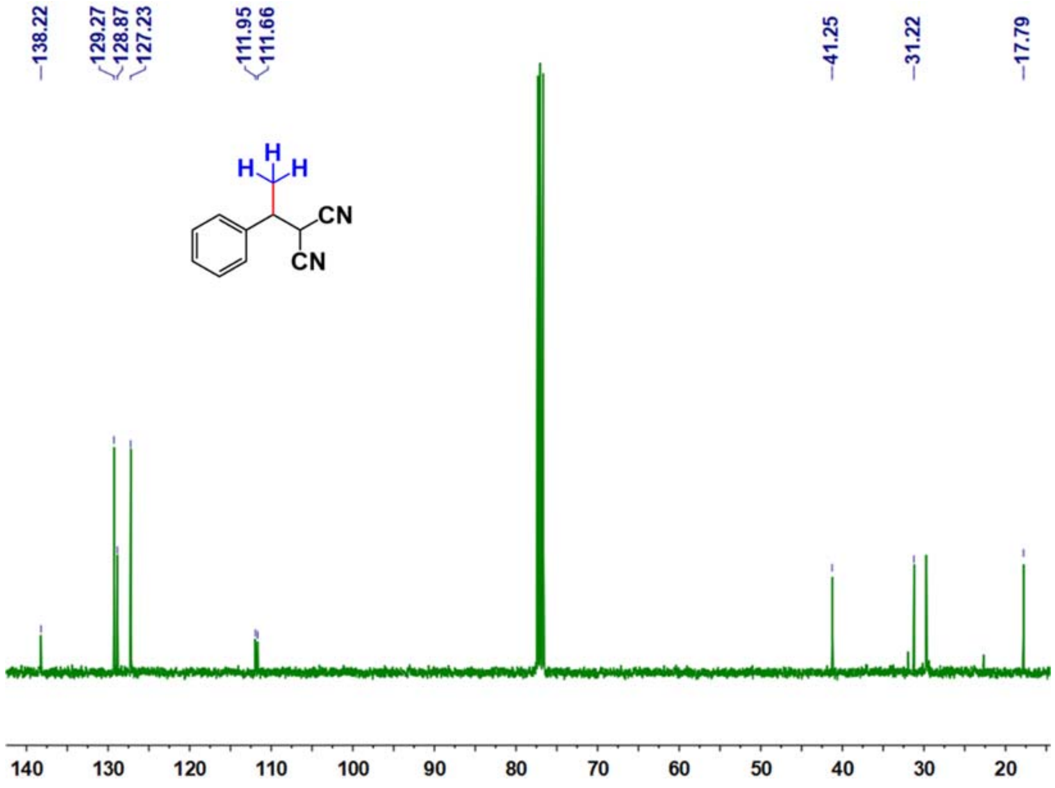

3j: 2−(2−Ethoxy−1−phenylethyl)malononitrile

White oil. Eluent: CH_2Cl_2. ^1H NMR (400 MHz, $CDCl_3$) δ 7.48 – 7.37 (m, 3H), 7.33 (d, J = 7.2 Hz, 2H), 3.92 (t, J = 13.7 Hz, 1H), 3.18 – 3.09 (m, 1H), 2.17 – 1.94 (m, 2H), 0.93 (t, J = 7.3 Hz, 3H); ^{13}C NMR (101 MHz, $CDCl_3$) δ 136.63, 129.27, 128.86, 127.91, 112.02, 48.19, 30.01, 25.43, 11.64.

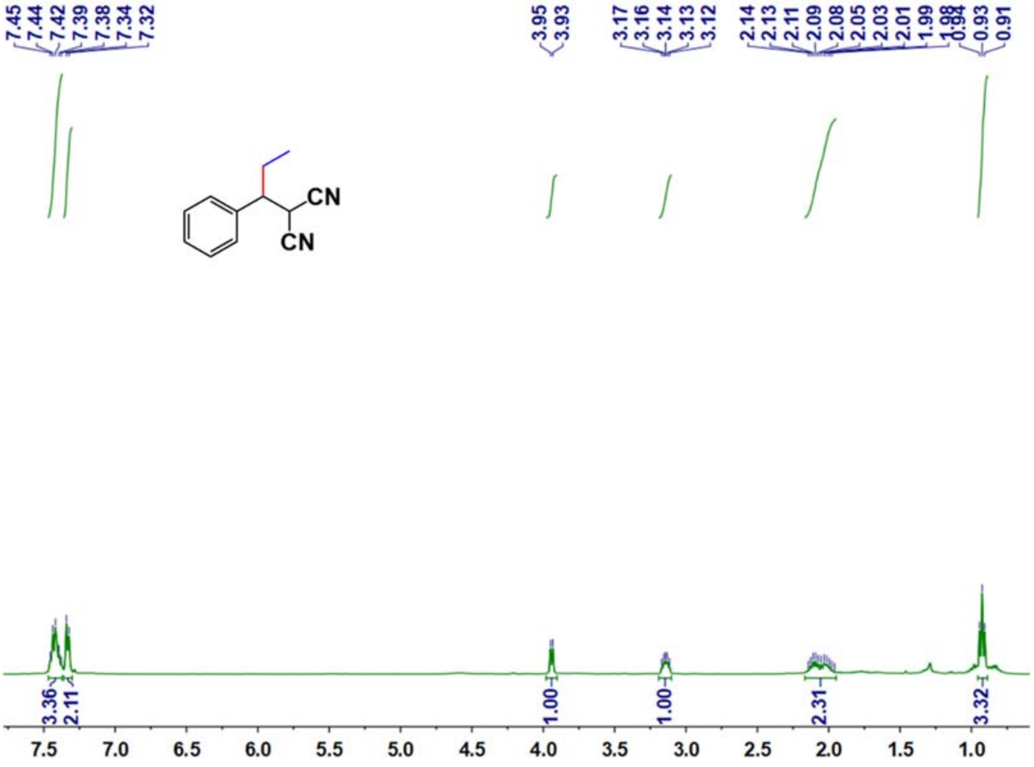

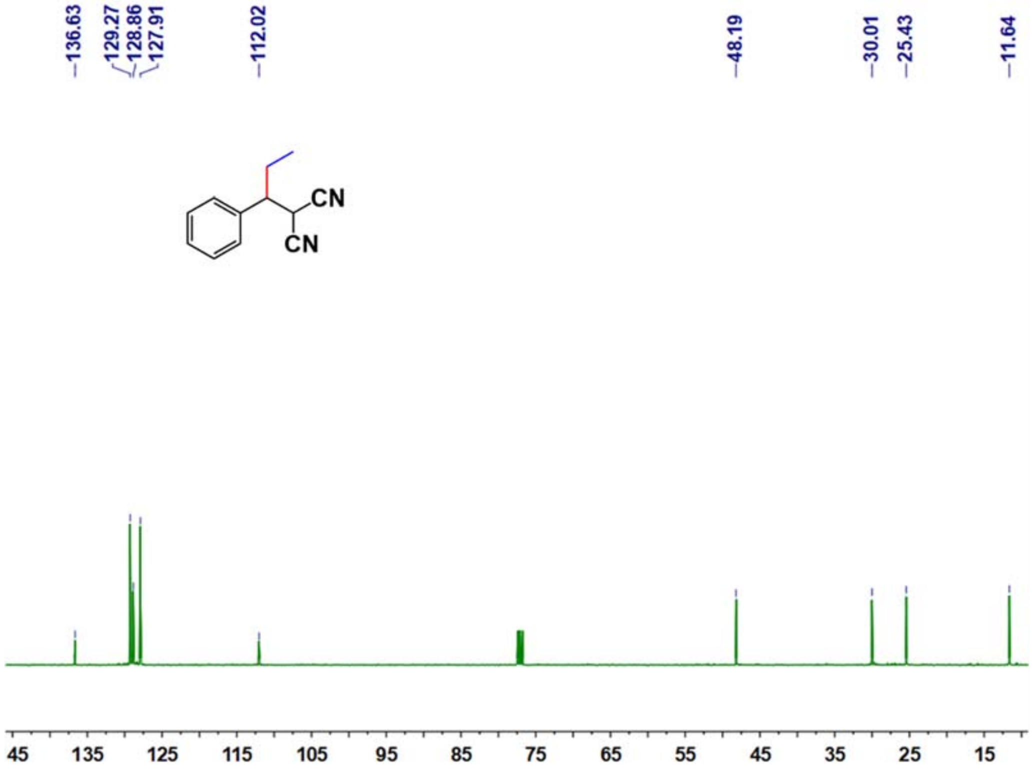

3k: 2-(2-methyl-1-phenylpropyl) malononitrile (3k-1) and 2-(1-phenylbutyl) malononitrile (3k-2)

Pale-yellow oil. Eluent: CH_2Cl_2. (**3k-1**:**3k-2** = 1:1.3). **3k-1:** ^{1}H NMR (400 MHz, $CDCl_3$) δ 7.47 – 7.15 (m, 5H), 3.83 (d, J = 6.3 Hz, 1H), 3.14 (dd, J = 15.1, 6.7 Hz, 1H), 1.93 – 1.85 (m, 2H), 1.25 – 1.11 (m, 2H), 0.84 (t, J = 7.3 Hz, 3H). **3k-2:** ^{1}H NMR (400 MHz, $CDCl_3$) δ 7.47 – 7.15 (m, 5H), 4.11 (d, J = 5.7 Hz, 1H), 2.77 (dd, J = 9.7, 5.7 Hz, 1H), 2.30 (ddt, J = 13.2, 9.7, 6.6 Hz, 1H), 1.06 (t, J = 6.2 Hz, J = 6.4 Hz, 3H), 0.75 (d, J = 6.6 Hz, 3H).

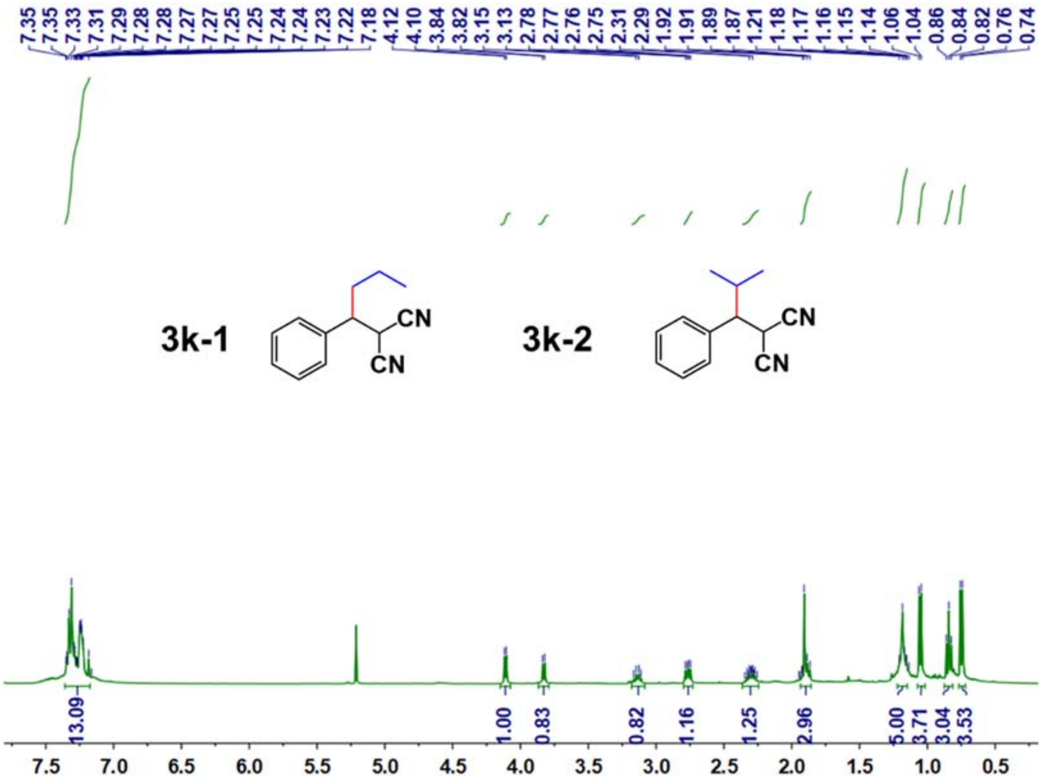

3l: 2-(2-methyl-1-phenylbutyl)malononitrile (3l-1) and 2-(1-phenylpentyl)malononitrile (3l-2):

pale-yellow oils. Eluent: CH_2Cl_2. (**3l-1**: **3l-2** = 2.1:1). **3l-1:** ^{1}H NMR (400 MHz, $CDCl_3$) δ 7.47 – 7.30 (m, 5H), 3.90 (d, J = 6.0 Hz, 1H), 3.22 (dd, J = 14.6, 7.3 Hz, 1H), 2.11 – 1.97 (m, 2H), 1.40 – 1.21 (m, 4H), 0.93 – 0.82 (m, 3H). **3l-2:** ^{1}H NMR (400 MHz, $CDCl_3$) δ 7.47 – 7.30 (m, 5H), 4.19 (d, J = 5.8 Hz, 1H),, [3.08 – 3.01 (m), 2.93 (dd, J = 10.0, 5.3 Hz, 1H)], 2.28 – 2.15 (m, 1H), [1.69 – 1.55 (m), 1.42 – 1.19 (m), 2H], [1.15 (d, J = 6.6 Hz), 0.92 – 0.82 (m), 3H], [1.03 (t, J = 7.0 Hz), 0.92 – 0.82 (m), 3H].

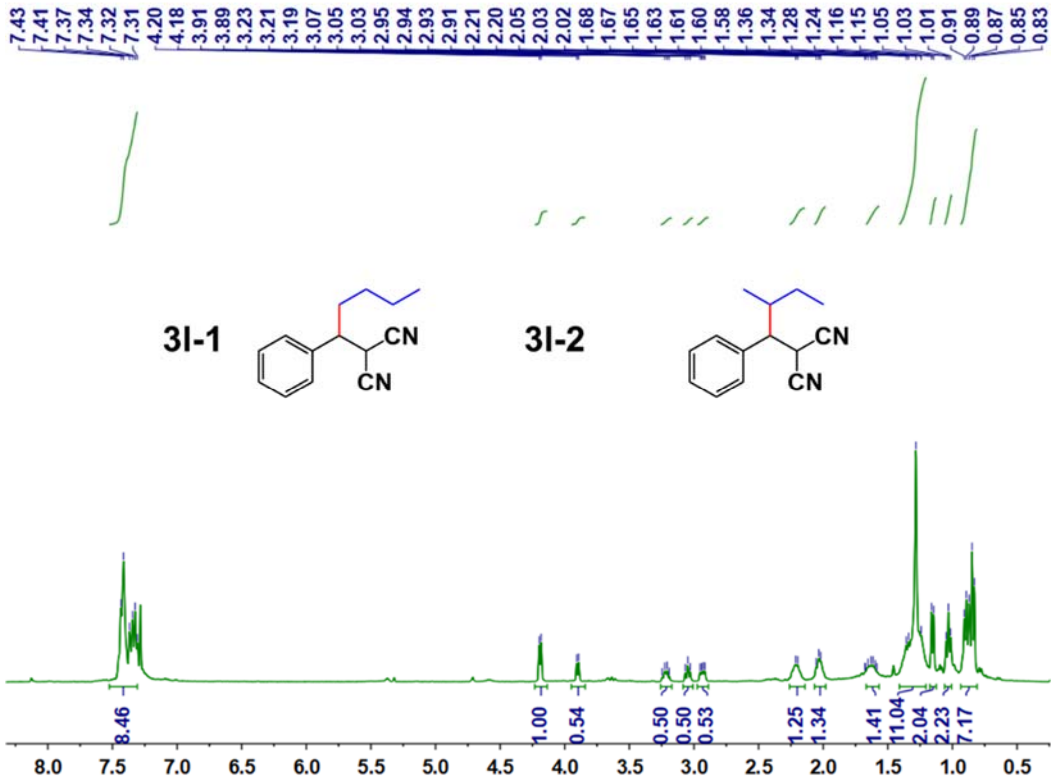

8. The ^{1}H/ ^{13}C NMR of Decarboxylative Functionalization Products

5a: 2−(2−Cyclohexyl−1−phenylethyl)malononitrile

White oil. Eluent: CH_2Cl_2. ^{1}H NMR (400 MHz, $CDCl_3$) δ 7.41−7.35 (m, 3H), 7.32−7.30 (m, 2H), 4.19−4.18 (d, J = 4.0 Hz, 1H), 2.89−2.87 (m, 1H), 2.04−1.99 (m, 1H), 1.93−1.91 (m, 1H), 1.85−1.83 (m, 1H), 1.69−1.64 (m, 2H), 1.48−1.37 (m, 1H), 1.39−1.37 (m, 1H), 1.20−1.04 (m, 3H), 0.85−0.79 (m, 1H); ^{13}C NMR (101 MHz, $CDCl_3$) δ 136.75, 129.11, 128.72, 128.32, 112.25, 111.99, 52.32, 39.26, 31.19, 30.57, 27.12, 25.85, 25.82, 25.73.

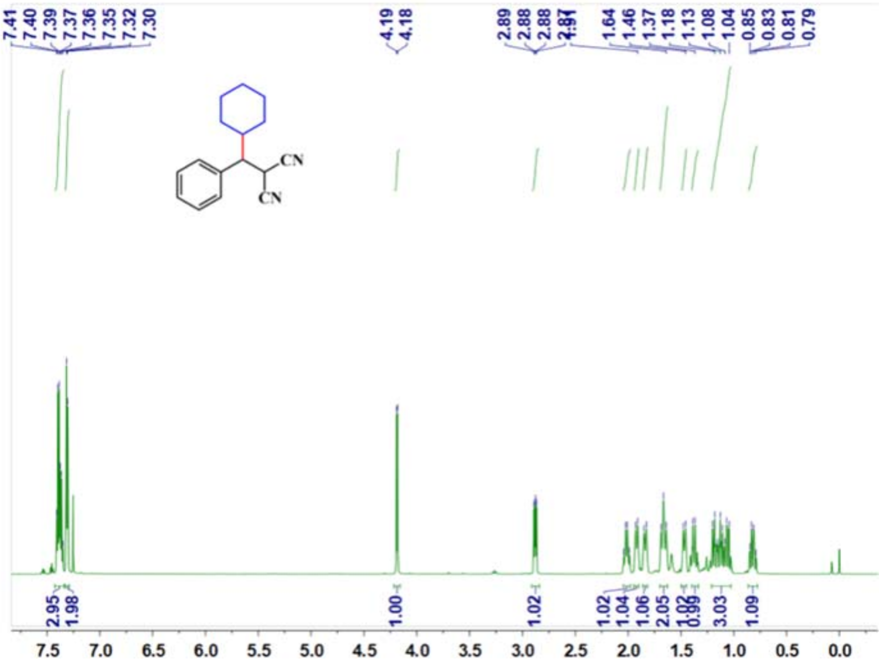

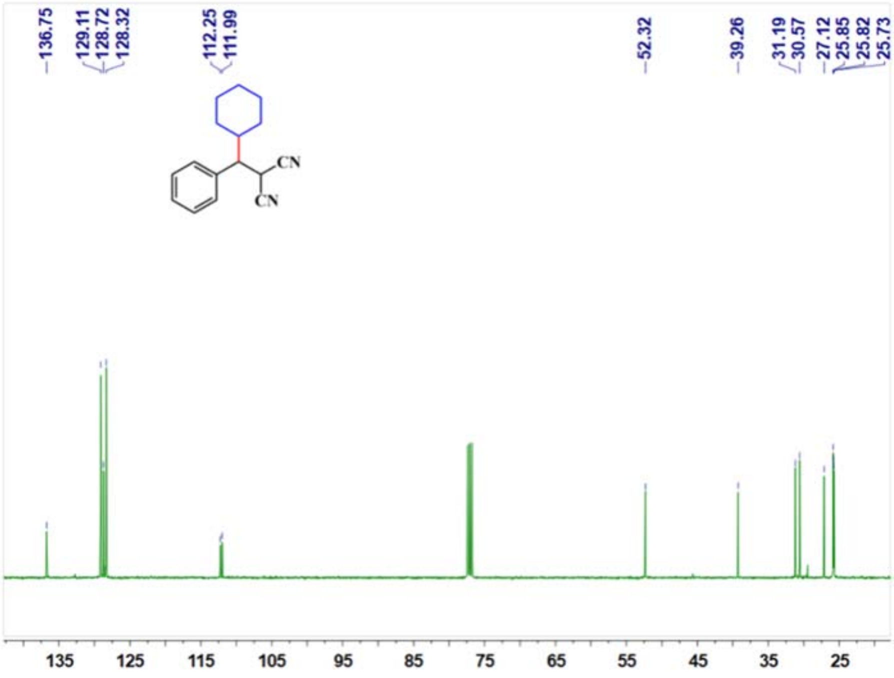

5b: 2−(2−Cyclopentyl−1−phenylethyl)malononitrile

White oil. Eluent: CH_2Cl_2. ^1H NMR (400 MHz, $CDCl_3$) δ 7.42−7.36 (m, 5H), 4.09−4.08 (d, J = 4.0 Hz, 1H), 2.94−2.90 (m, 1H), 2.58−2.52 (m, 1H), 2.05−2.02 (m, 1H), 1.75−1.59 (m, 5H), 1.31−1.25 (m, 1H), 1.08−1.01 (m, 1H); ^{13}C NMR (101 MHz, $CDCl_3$) δ 137.37, 129.07, 128.76, 128.19, 112.08, 111.83, 52.28, 42.41, 31.67, 31.61, 29.16, 25.47.

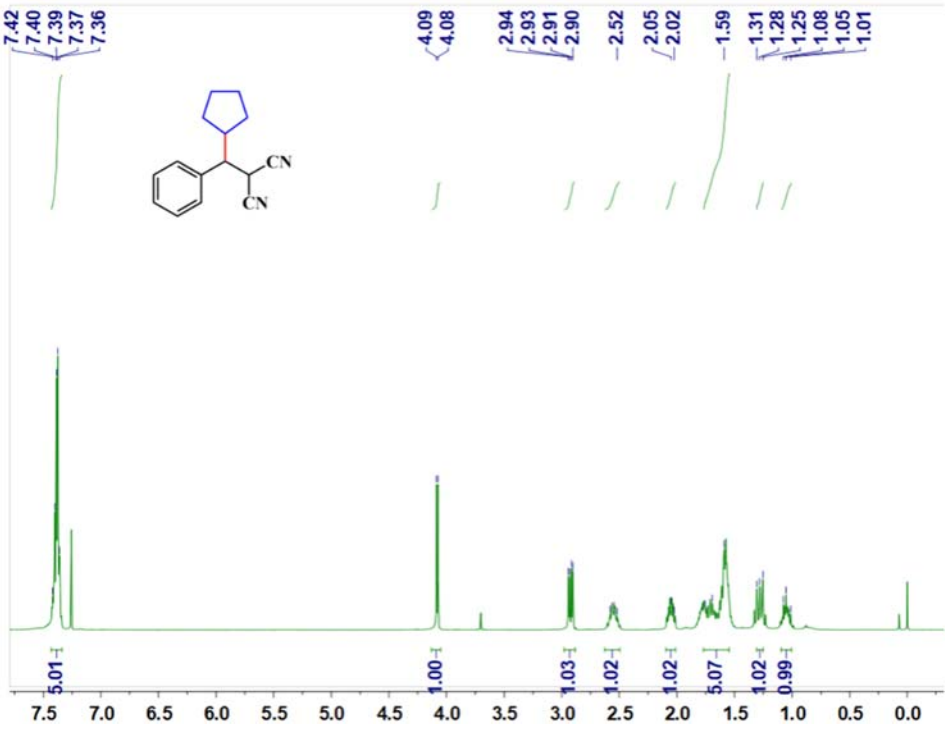

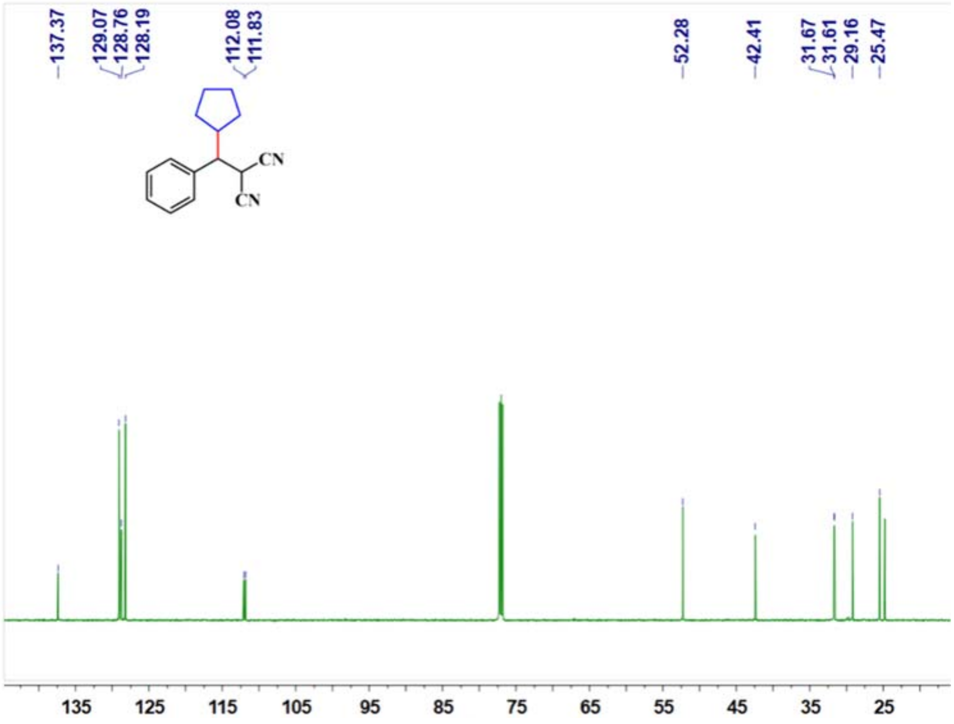

5c: 2−(2−Cyclobutyl−1−phenylethyl)malononitrile

White oil. Eluent: CH_2Cl_2. ^{1}H NMR (400 MHz, $CDCl_3$) δ 7.44−7.38 (m, 3H), 7.34−7.32 (m, 2H), 3.92−3.91 (d, J = 4.0 Hz, 1H), 3.22−3.18 (m, 1H), 3.08−3.03 (m, 1H), 2.41−2.34 (m, 1H), 2.00−1.84 (m, 4H), 1.66−1.59 (m, 1H); ^{13}C NMR (101 MHz, $CDCl_3$) δ 135.57, 129.12, 128.83, 128.11, 112.00, 111.68, 52.73, 37.31, 27.88, 27.65, 27.04, 17.51.

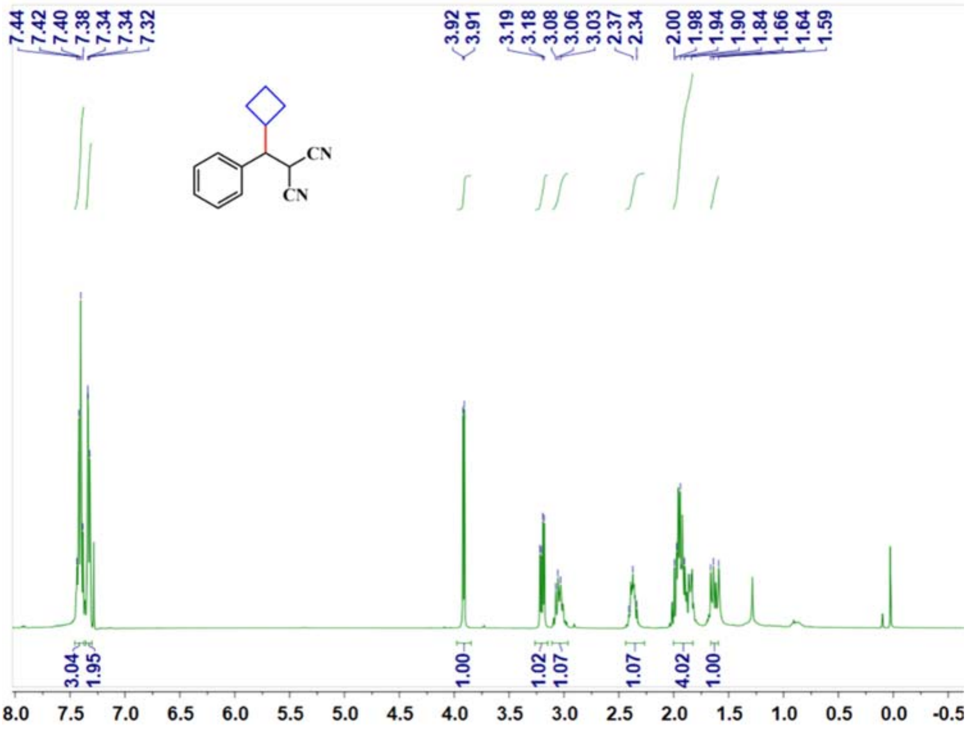

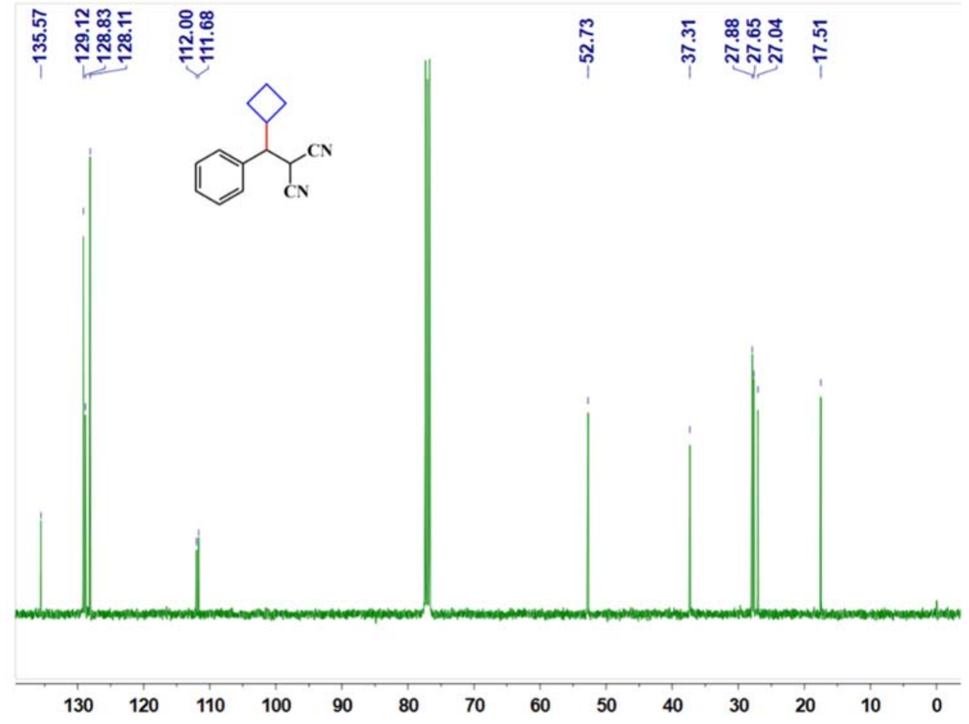

5d: 2−[2−(4−(*N*−boc−piperidine)−yl)−1−phenylethyl]malononitrile

White oil. Eluent: CH_2Cl_2. ^1H NMR (400 MHz, $CDCl_3$) δ 7.44–7.39 (m, 3H), 7.33–7.31 (m, 2H), 4.24−4.04 (m, 3H), 2.92−2.88 (m, 1H), 2.82−2.59 (m, 2H), 2.21−2.13 (m, 1H), 1.88−1.85 (m, 1H), 1.43 (s, 9H), 1.29−1.26 (m, 2H), 1.07−1.02 (m, 1H); ^{13}C NMR (101 MHz, $CDCl_3$) δ 154.52, 135.94, 129.37, 129.10, 128.17, 111.80, 111.71, 79.77, 51.73, 38.09, 30.25, 29.82, 28.40, 27.04.

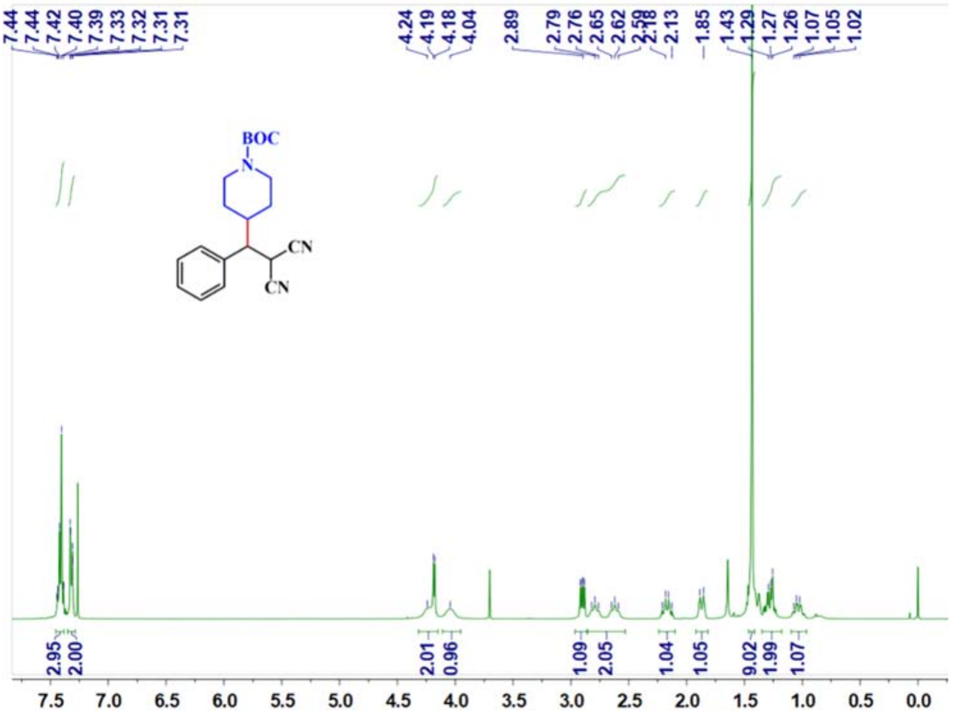

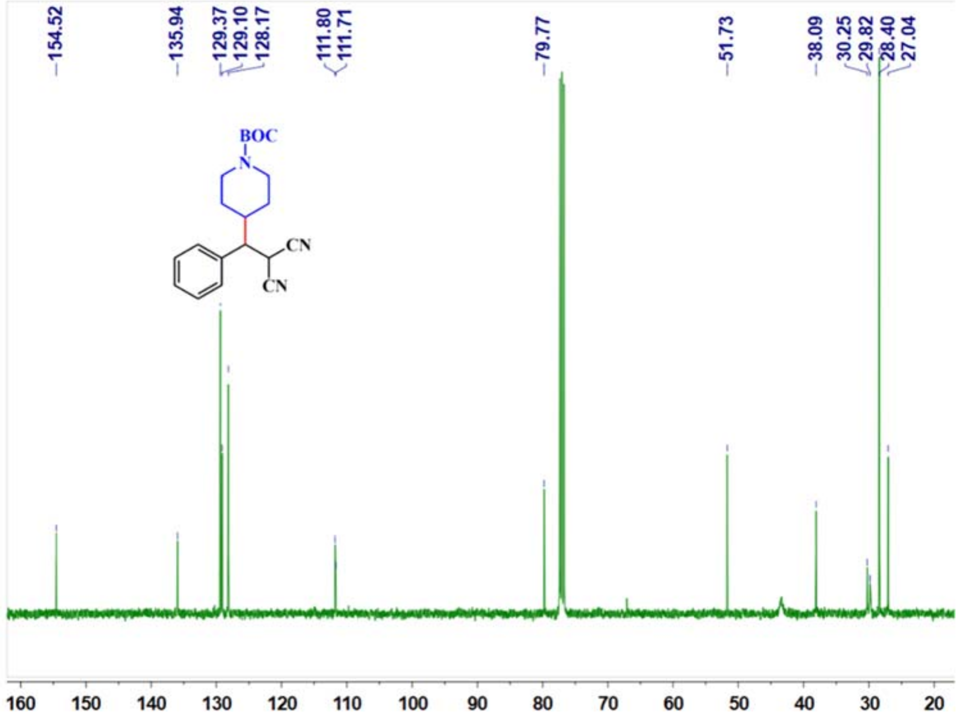

5e: 2−((2,3−Dihydro−1*H*−inden−2−yl)(phenyl)methyl)malononitrile

White oil. Eluent: CH_2Cl_2. ^{1}H NMR (400 MHz, $CDCl_3$) δ 7.45–7.41 (m, 5H), 7.25−7.23 (m, 1H), 7.19−7.12 (m, 2H), 7.09−7.07 (m, 1H), 4.15–4.14 (d, J = 4.0, 1H), 3.34−3.23 (m, 3H), 2.88−2.78 (m, 2H), 2.56−2.50 (m, 1H); ^{13}C NMR (101 MHz, $CDCl_3$) δ 141.71, 141.21, 136.76, 129.39, 129.12, 128.18, 126.92, 126.82, 124.50, 124.37, 111.83, 111.63, 51.64, 42.51, 38.32, 37.75, 28.98.

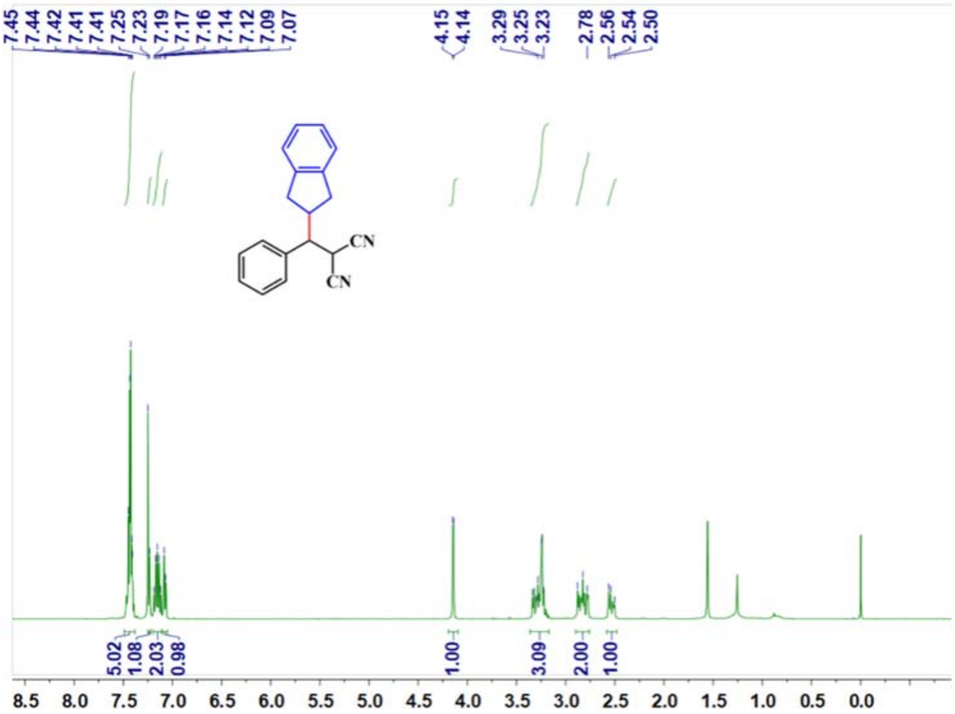

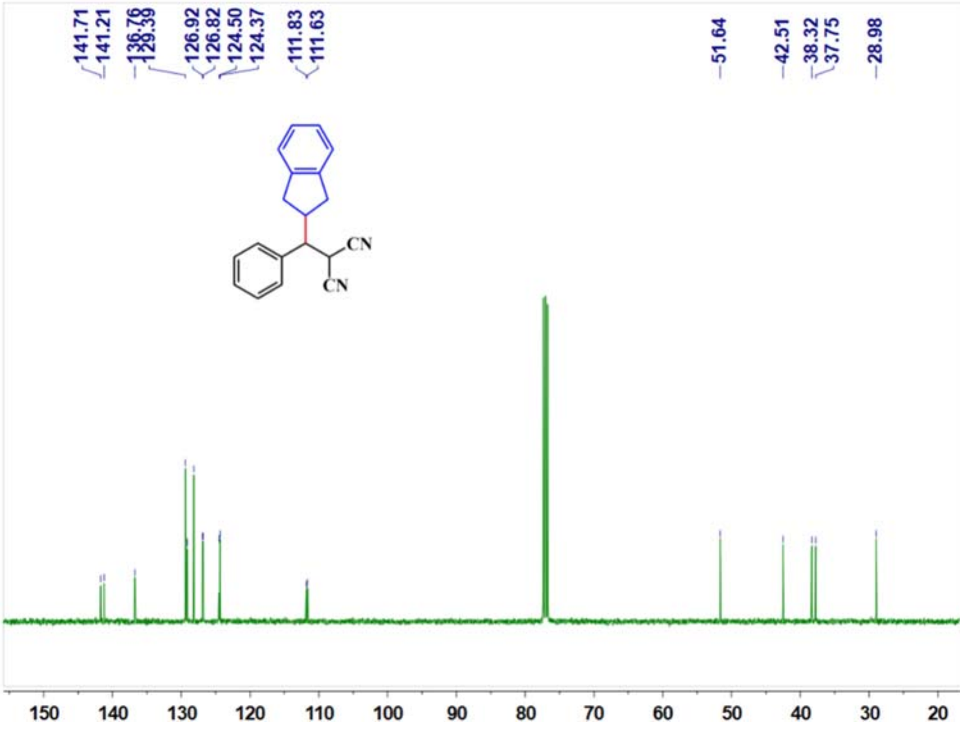

5f: 2−[2−(4−(Tetrahydropyran)−yl)−1−phenylethyl]malononitrile

White oil. Eluent: CH_2Cl_2. ^{1}H NMR (400 MHz, $CDCl_3$) δ 7.44–7.39 (m, 3H), 7.35−7.33 (m, 2H), 4.17−4.16 (m, 1H), 4.09−4.06 (m, 1H), 3.91−3.87 (m, 1H), 3.51−3.45 (m, 1H), 3.36−3.30 (m, 1H), 2.92−2.88 (m, 1H), 2.31−2.23 (m, 1H), 1.82−1.79 (m, 1H), 1.53−1.42 (m, 1H), 1.32−1.19 (m, 3H); ^{13}C NMR (101 MHz, $CDCl_3$) δ 135.76, 129.36, 129.10, 128.23, 111.80, 111.67, 67.53, 67.11, 51.97, 37.00, 31.07, 30.67, 26.81.

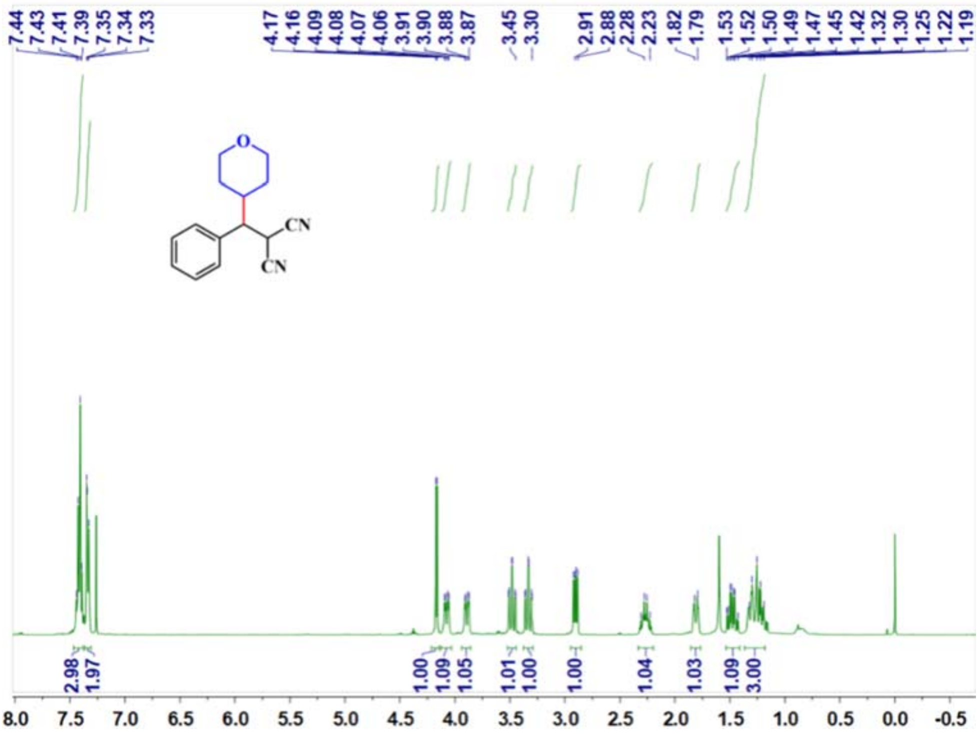

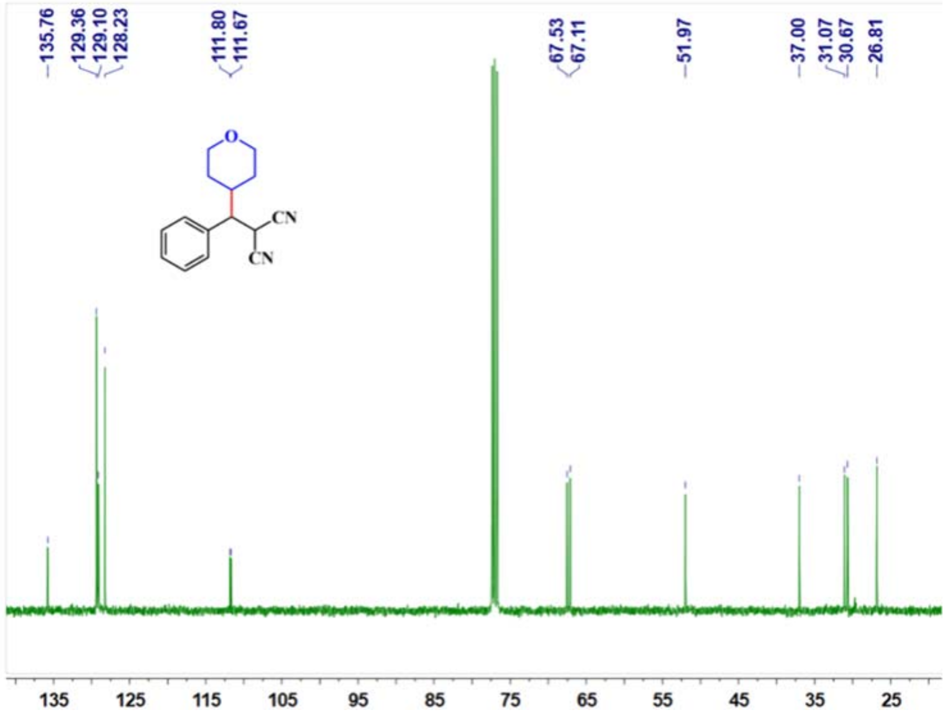

5g: 2−(2−Pentyl−1−phenylethyl)malononitrile

White oil. Eluent: CH_2Cl_2. ^1H NMR (400 MHz, $CDCl_3$) δ 7.43−7.37 (m, 3H), 7.32−7.30 (m, 2H), 3.88−3.87 (d, J = 4.0 Hz, 1H), 3.22−3.17 (m, 1H), 2.02−1.96 (m, 2H), 1.28−1.23 (m, 6H), 0.86−0.83 (m, 3H); ^{13}C NMR (101 MHz, $CDCl_3$) δ 136.85, 129.29, 128.87, 127.83, 111.95, 46.61, 32.07, 31.28, 30.27, 26.63, 22.30, 13.90.

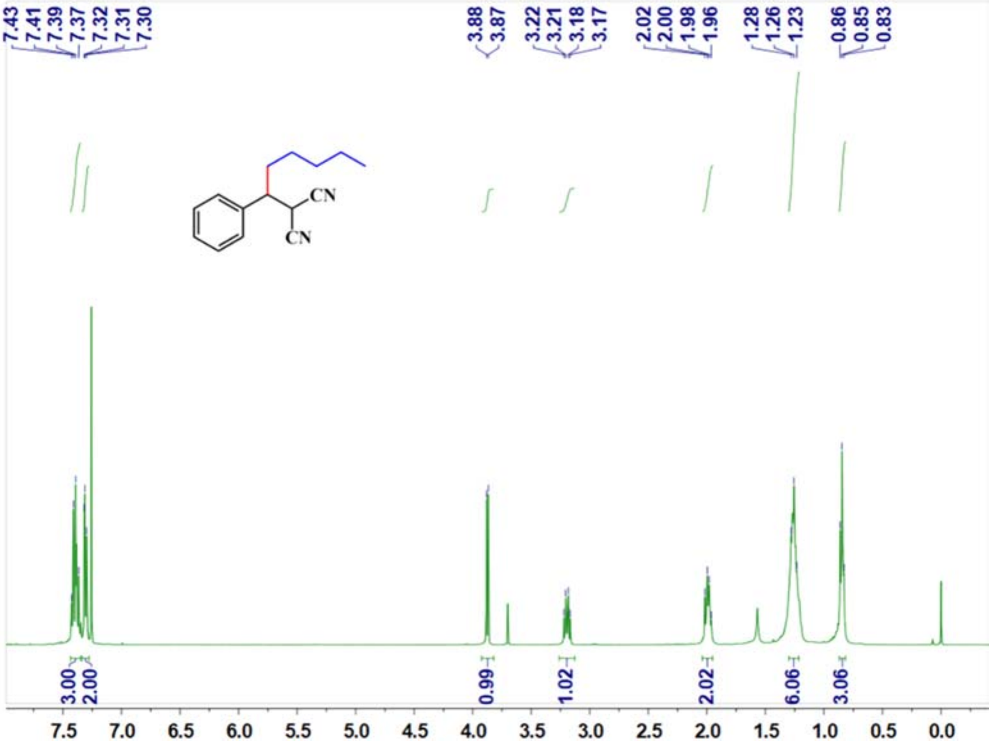

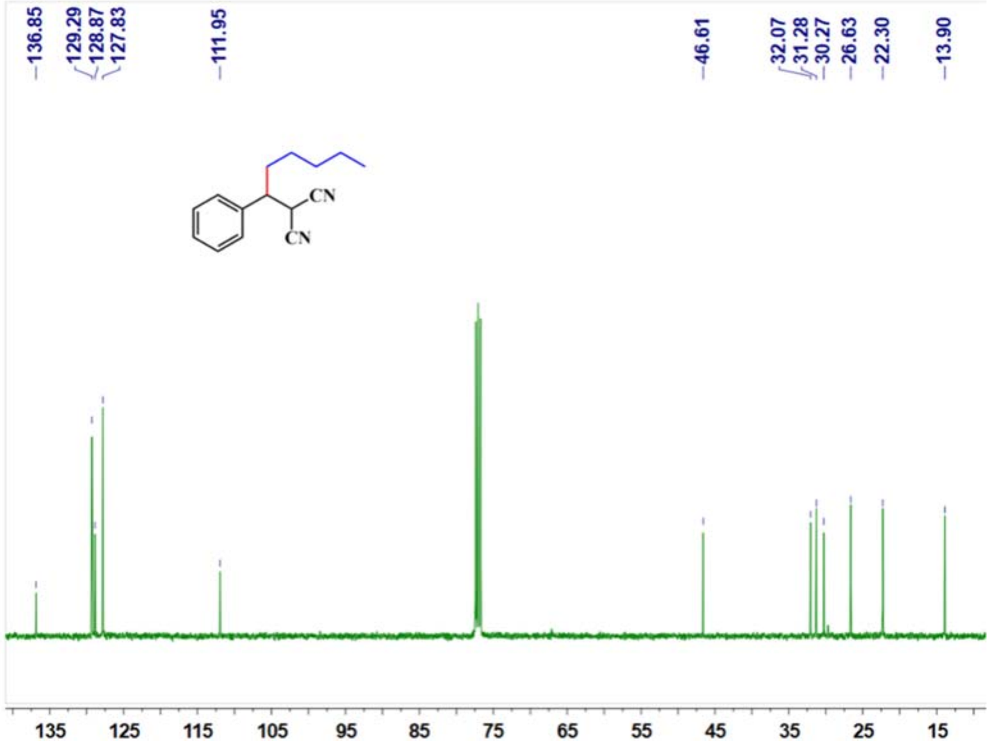

5h: 2−(2−Isobutyl−1−phenylethyl)malononitrile

White oil. Eluent: CH_2Cl_2. ^{1}H NMR (400 MHz, $CDCl_3$) δ 7.42−7.36 (m, 3H), 7.32−7.31 (m, 2H), 3.84−3.83 (d, J = 4.0 Hz, 1H), 3.31−3.28 (m, 1H), 2.05−2.00 (m, 1H), 1.75−1.71 (m, 1H), 1.45−1.40 (m, 1H), 0.92−0.89 (m, 6H); ^{13}C NMR (100 MHz, $CDCl_3$) δ 136.67, 129.31, 128.90, 127.89, 111.92, 111.89, 44.68, 40.88, 30.61, 25.23, 23.34, 21.08.

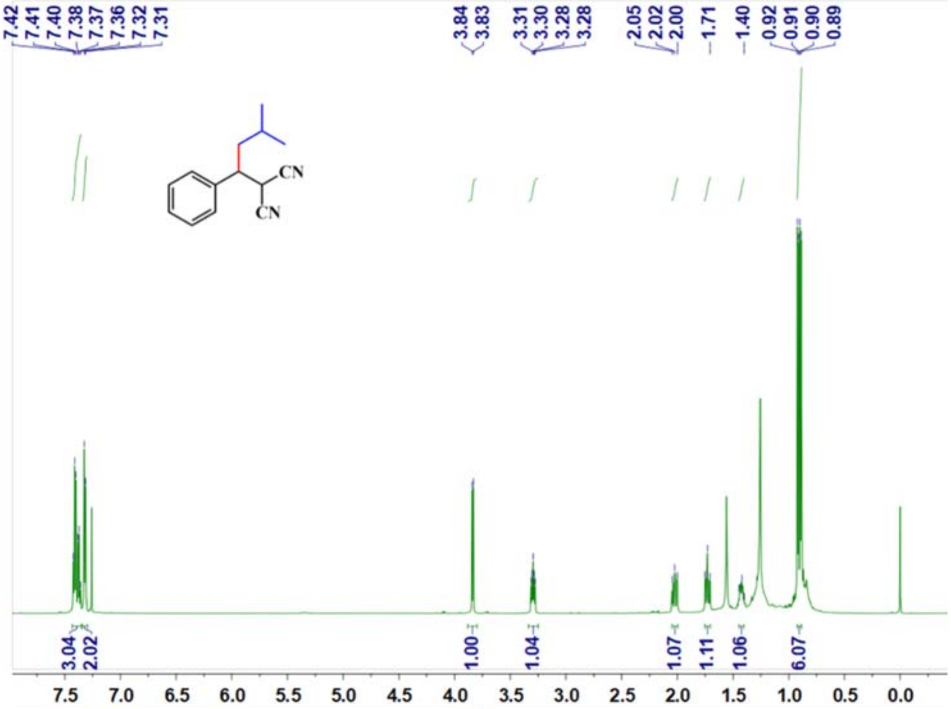

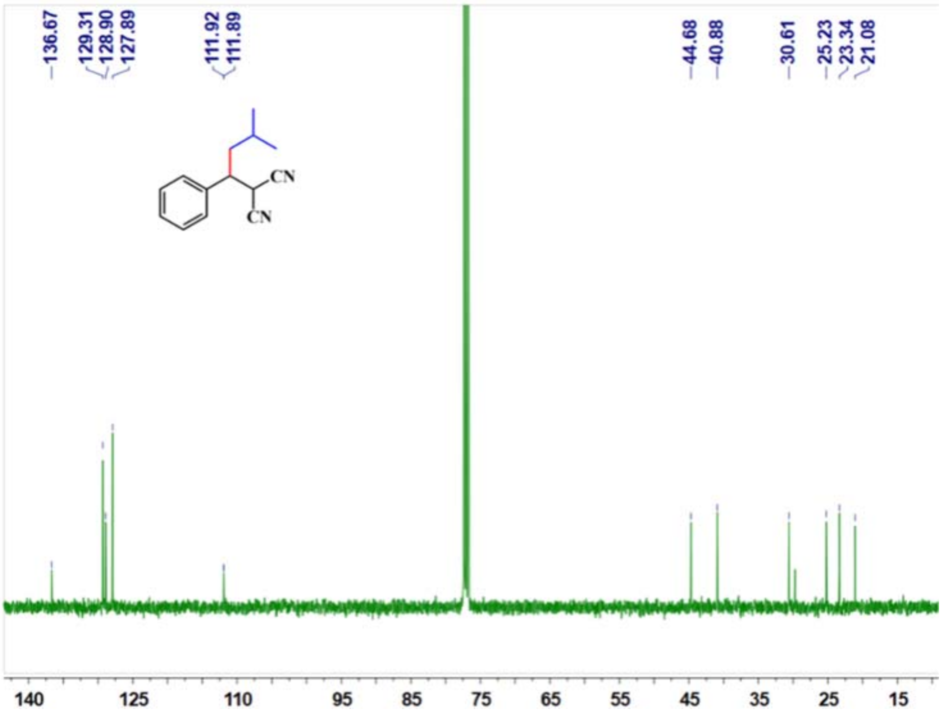

5i: 2−(2−Heptyl−1−phenylethyl)malononitrile

White oil. Eluent: CH_2Cl_2. ^{1}H NMR (400 MHz, $CDCl_3$) δ 7.42−7.36 (m, 3H), 7.31−7.30 (m, 2H), 3.88−3.87 (d, J = 4.0 Hz, 1H), 3.21−3.18 (m, 1H), 2.01−1.98 (m, 2H), 1.29−1.21 (m, 10H), 0.87−0.84 (t, J = 12.0 Hz, 3H); ^{13}C NMR (101 MHz, $CDCl_3$) δ 136.87, 129.28, 128.86, 127.82, 111.92, 46.64, 31.64, 29.08, 28.90, 26.96, 22.54, 14.01.

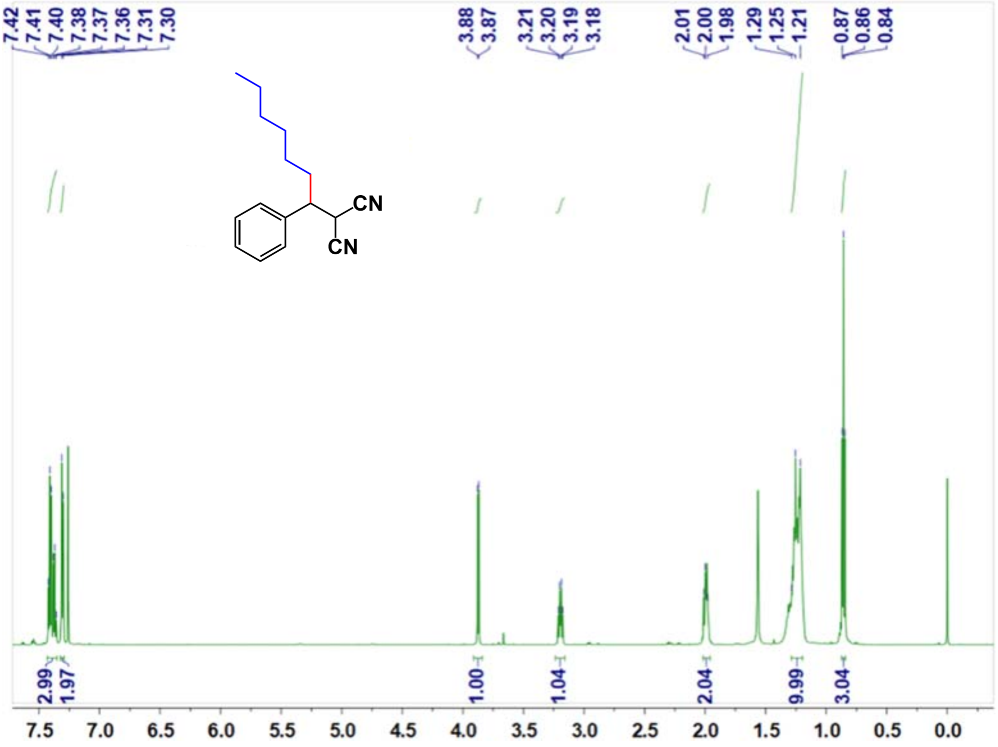

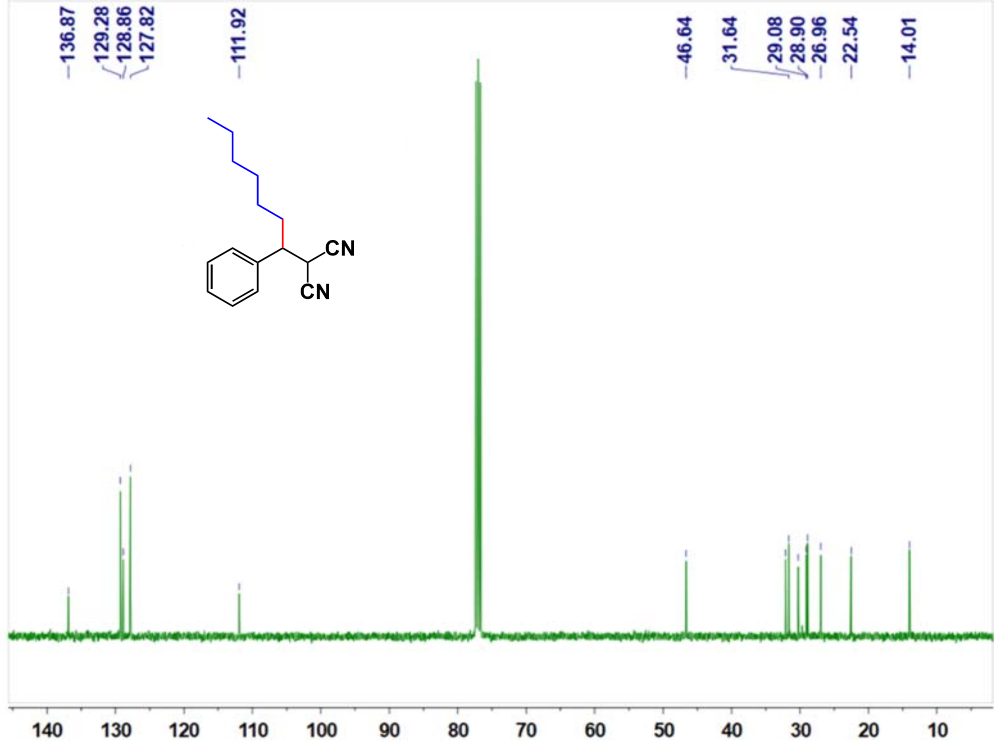

5j: 2−(2−Adamantylmethyl−1−phenylethyl)malononitrile

hite oil. Eluent: CH_2Cl_2. ^{1}H NMR (400 MHz, $CDCl_3$) δ 7.41–7.35 (m, 5H), 3.77–3.76 (d, J = 4.0 Hz, 1H), 3.42−3.37 (m, 1H), 2.08−2.02 (m, 1H), 1.89 (s, 3H), 1.67−1.63 (m, 4H), 1.56 (s, 3H), 1.43−1.33 (m, 6H); ^{13}C NMR (101 MHz, $CDCl_3$) δ 138.85, 129.21, 128.73, 128.01, 112.16, 112.03, 46.19, 42.64, 42.09, 36.70, 33.43, 33.10, 32.19, 29.71, 28.41.

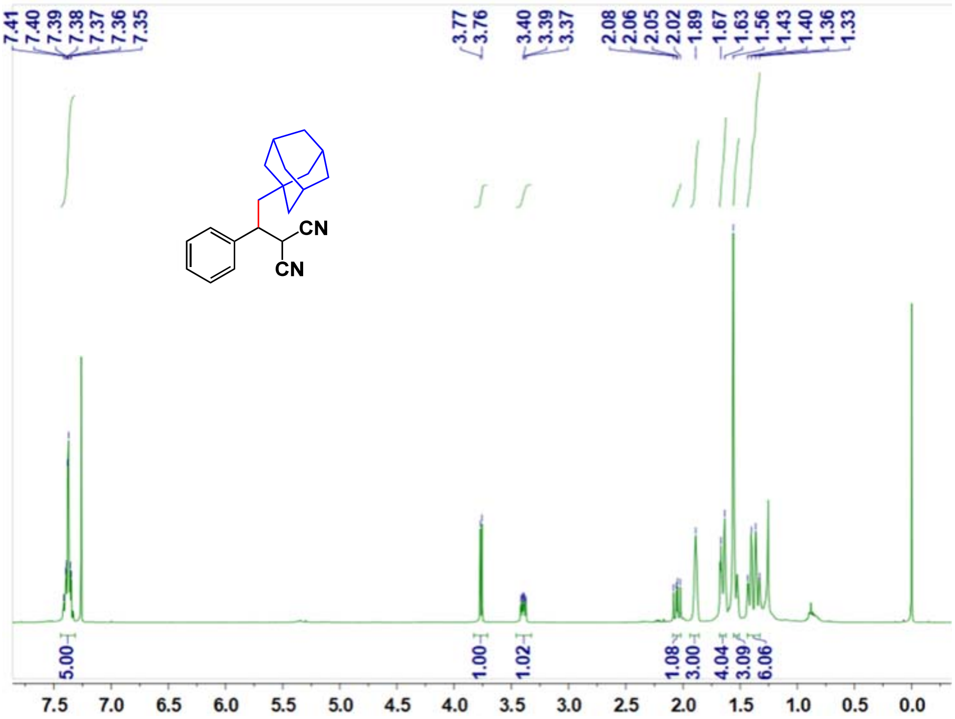

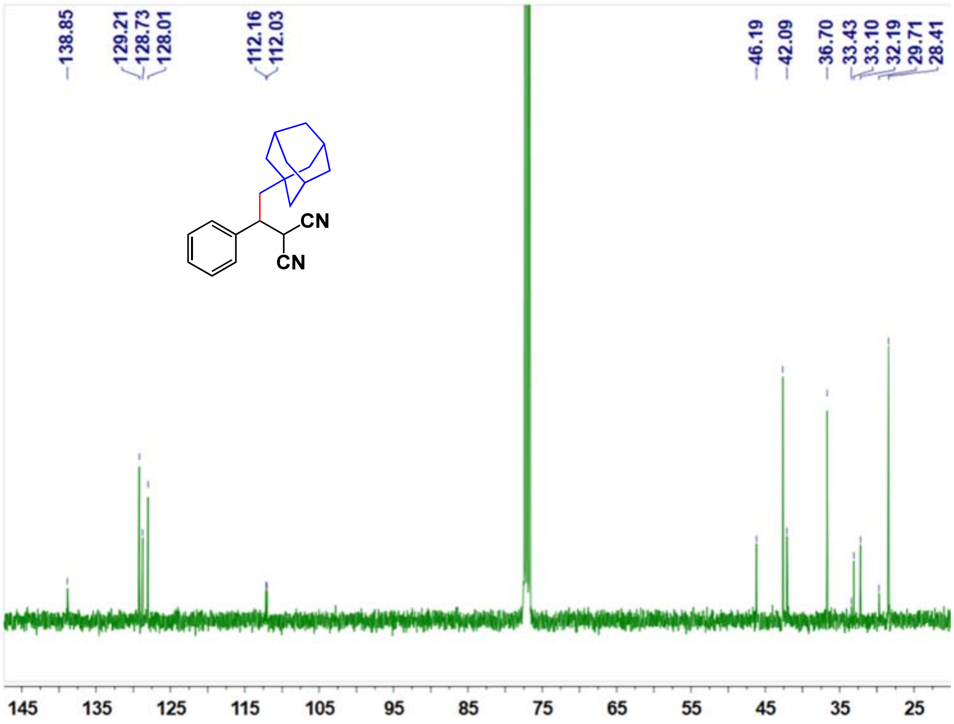

5k: 2−(2−Benzoyl−1−phenylethyl)malononitrile: White oil. Eluent: CH_2Cl_2. ^{1}H NMR (400 MHz, $CDCl_3$) δ 7.91–7.89 (m, 2H), 7.57−7.53 (m, 1H), 7.42−7.39 (m, 5H), 7.37−7.34 (m, 2H), 5.12−5.10 (d, J = 8.0 Hz, 1H), 4.55−4.53 (d, J = 8.0 Hz, 1H); ^{13}C NMR (101 MHz, $CDCl_3$) δ 193.03, 134.41, 133.91, 132.06, 130.07, 129.90, 129.25, 128.95, 128.61, 112.09, 111.51, 54.85, 26.82.

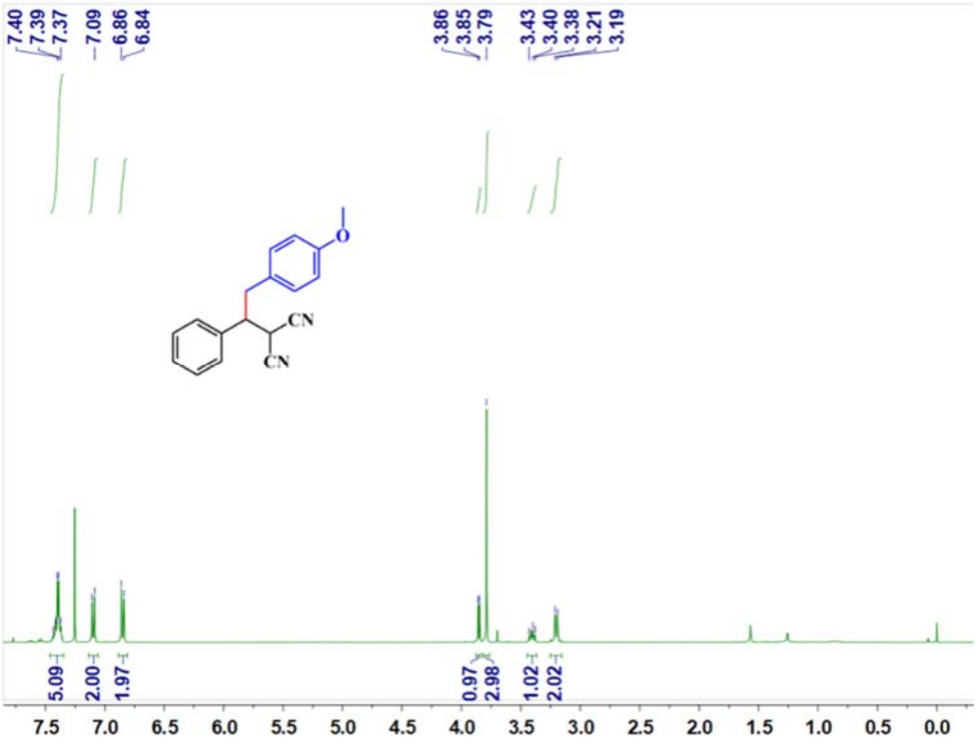

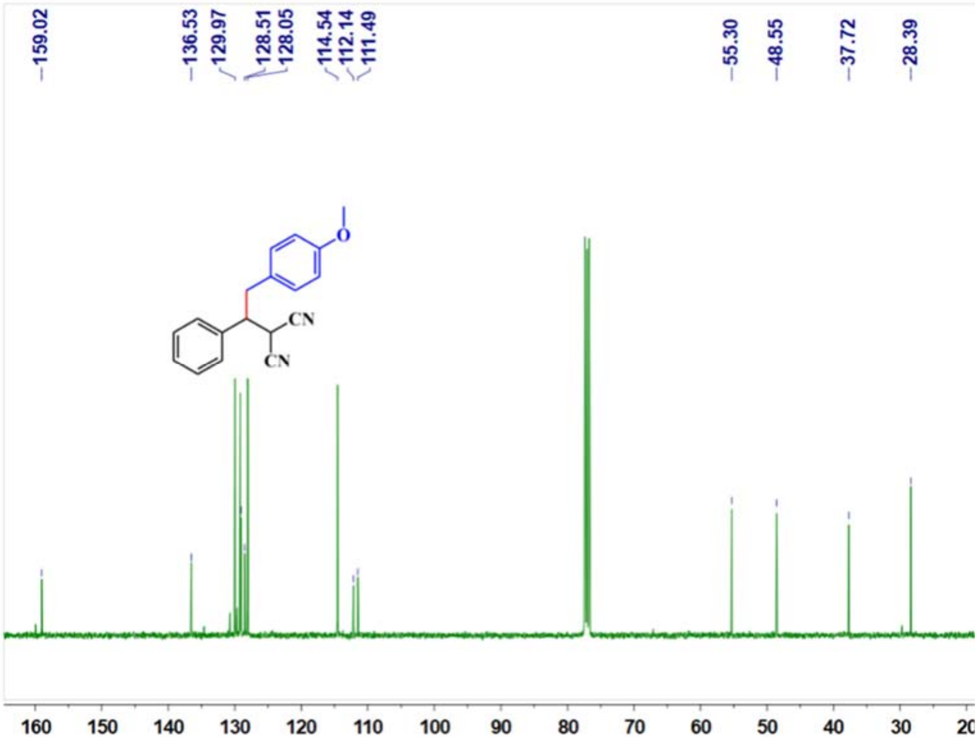

5l: 2−(2−(4−(Methoxyl)phenyl)−1−phenylethyl)malononitrile

White oil. Eluent: CH_2Cl_2. ^{1}H NMR (400 MHz, $CDCl_3$) δ 7.43−7.37 (m, 5H), 7.11−7.09 (m, 2H), 6.86−6.84 (m, 2H), 3.86−3.85 (d, J = 4.0 Hz, 1H), 3.79 (s, 3H), 3.43−3.38 (m, 1H), 3.21−3.19 (m, 2H); ^{13}C NMR (101 MHz, $CDCl_3$) δ 159.02, 136.53, 129.97, 128.51, 128.05, 114.54, 112.14, 111.49, 55.30, 48.55, 37.72, 28.39.

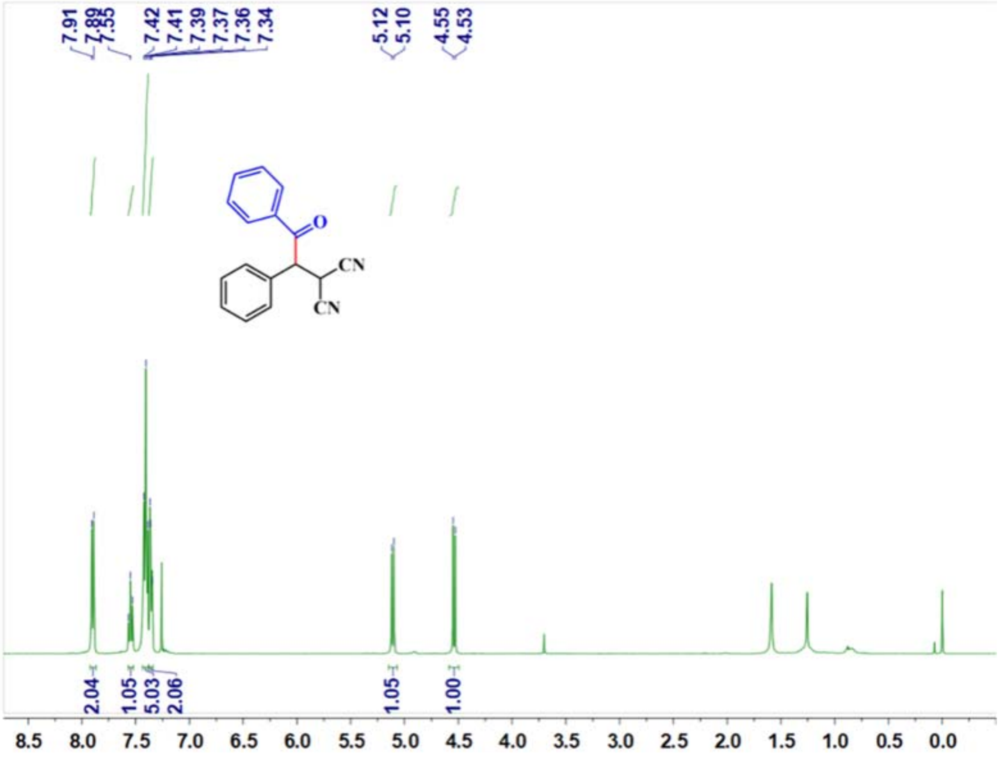

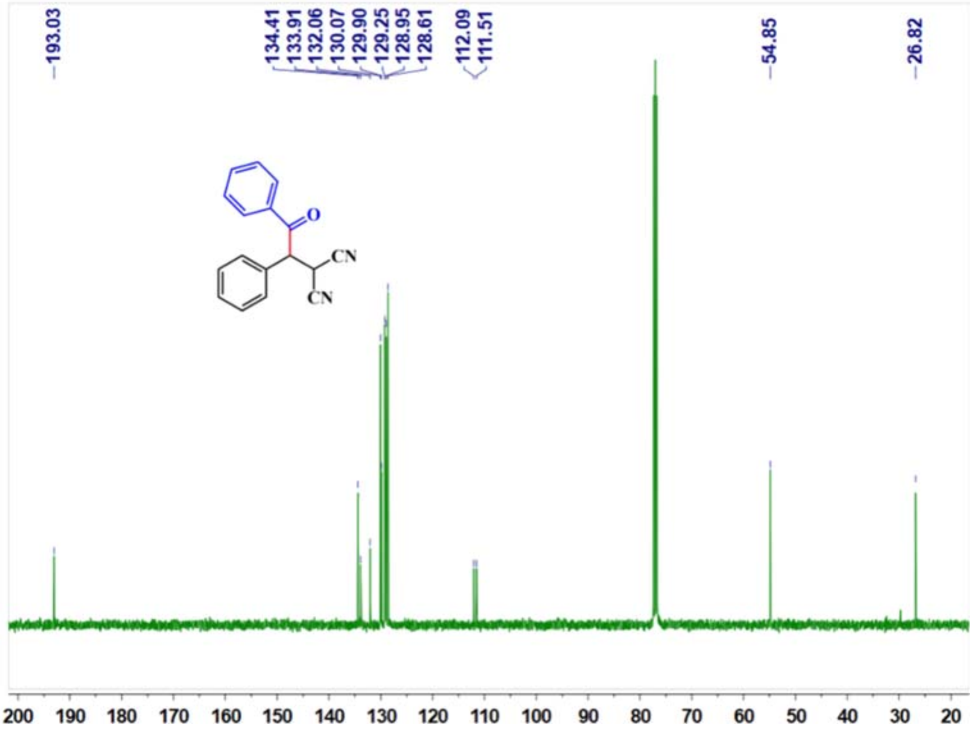

5m:

2−(2−(5−(2,5−Dimethylphenoxy)−2,2−dimethylpentyl)−1−phenylethyl)malononitrile

White oil. Eluent: CH_2Cl_2. ^{1}H NMR (400 MHz, $CDCl_3$) δ 7.41−7.38 (m, 5H), 7.02−7.00 (m, 1H), 6.68−6.59 (m, 2H), 4.25−4.23 (d, J = 8.0 Hz, 1H), 3.94−3.88 (m, 2H), 3.11−3.10 (d, J = 4, 1H), 2.30 (s, 3H), 2.14 (s, 3H), 1.83−1.76 (m, 2H), 1.60−1.52 (m, 2H), 1.19 (s, 3H), 1.07 (s, 3H); ^{13}C NMR (101 MHz, $CDCl_3$) δ 156.71, 136.60, 135.80, 128.72, 120.98, 113.31, 113.04, 111.96, 67.57, 55.12, 37.40, 37.21, 25.22, 23.96, 21.42, 15.85.

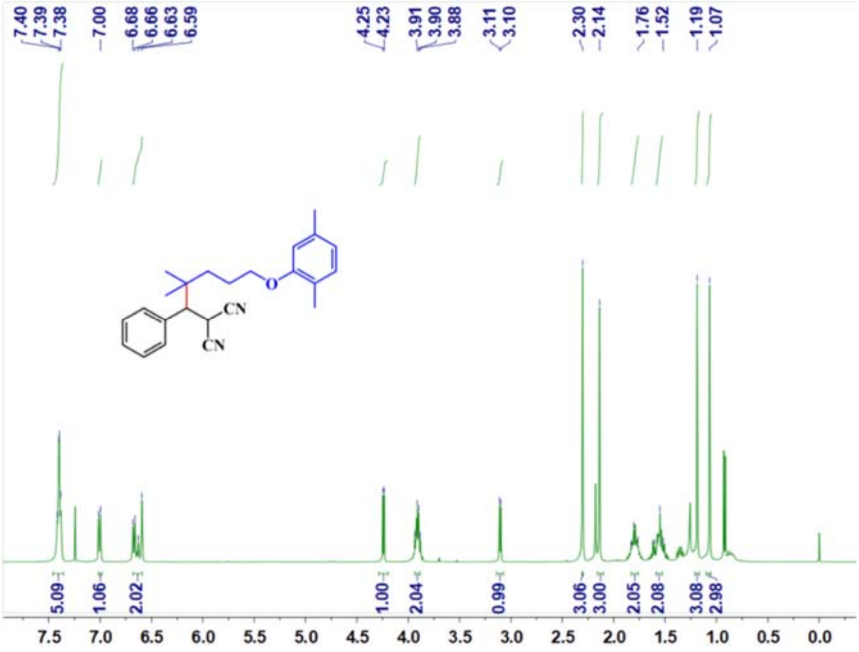

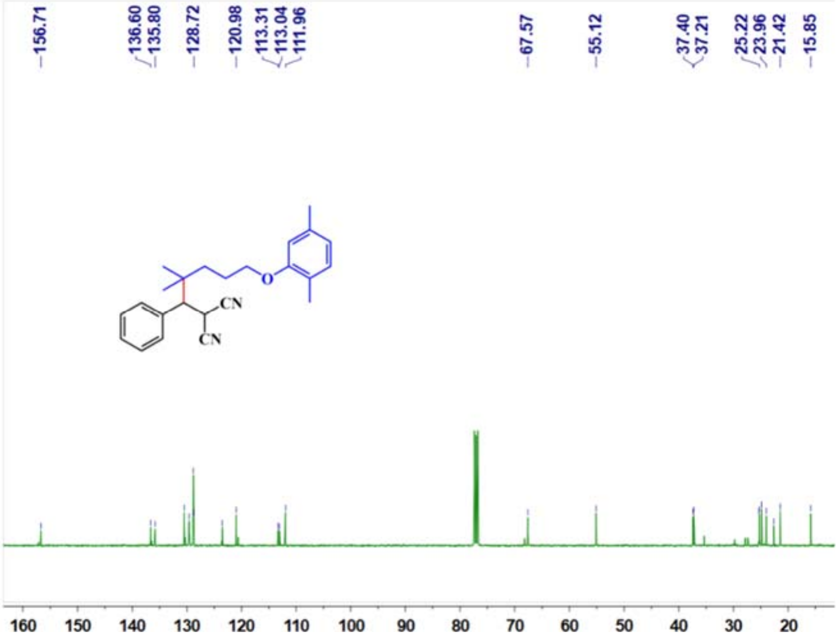

5n: 2−(2−(2−(4−Isobutylphenyl)−propyl)−1−phenylethyl)malononitrile

White oil. Eluent: CH_2Cl_2. ^1H NMR (400 MHz, $CDCl_3$) δ 7.52−7.43 (m, 5H), 7.28−7.26 (m, 2H), 7.21−7.19 (m, 2H), 3.62−3.61 (d, J = 4.0 Hz, 1H), 3.41−3.35 (m, 1H), 3.19−3.15 (m, 1H), 2,50−2.48 (d, J = 8.0 Hz, 2H), 1.92−1.85 (m, 1H), 1,13−1,12 (d, J = 4.0 Hz, 3H), 0.94−0.92 (d, J = 8.0 Hz, 6H); ^{13}C NMR (100 MHz, $CDCl_3$) δ 141.70, 139.55, 135.67, 130.34, 129.28, 128.58, 126.79, 112.28, 111.53, 53.74, 45.02, 41.65, 30.18, 28.73, 22.42, 20.58.

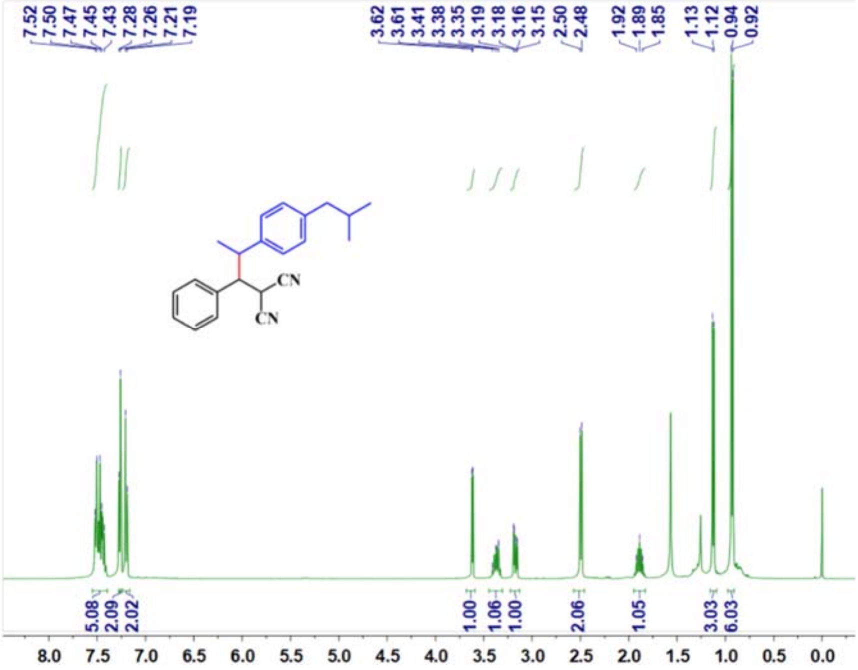

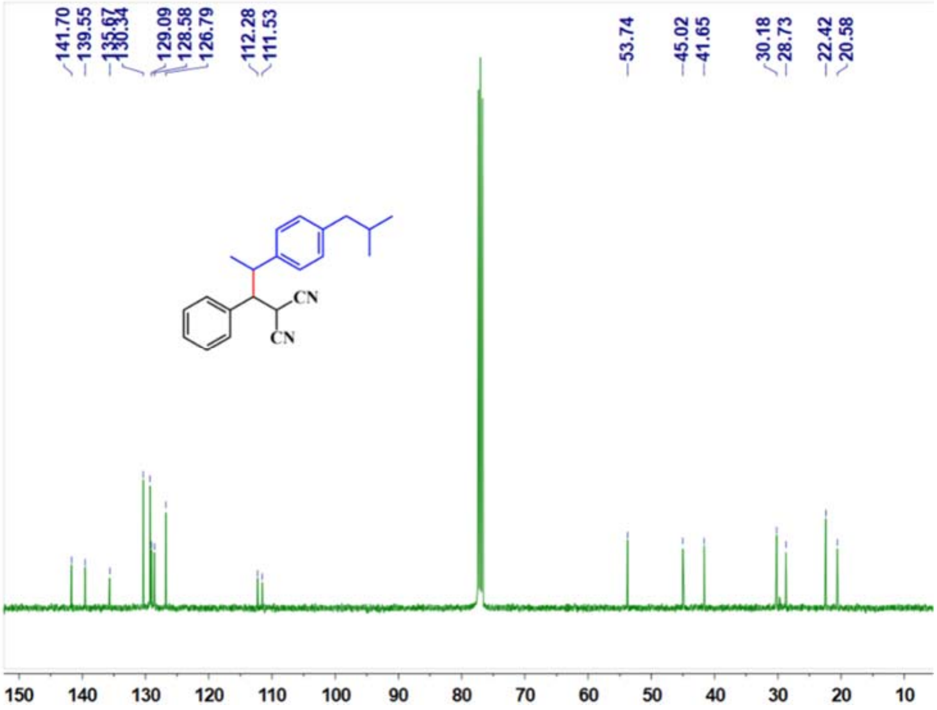

9. Quantification Method of Oxidation Product by Gas Chromatography

The standard sample was prepared by adding the same molar amount of the target oxidation product with 1,3,5-trimethoxybenzene as the internal standard, calibrations were carried out to obtain response factors (RF) of the target products compared to the internal standard substance 1,3,5-trimethoxybenzene. The RF value was obtained using the following equation:

$$\mathrm{RF} = \frac{\mathrm{Area}_{\ \mathrm{target\ product}}}{\mathrm{Area}_{\ \mathrm{internal\ standard}}}$$

In an analytical sample containing an equimolar amount of 1,3,5-trimethoxybenzene to the substrates, the yields of the target products were calculated according to the area of the chromatographic peak. The yields of products were calculated using the following equation:

$$\mathrm{Yield\ (\%)} = \frac{\mathrm{Area}_{\ \mathrm{target\ product}}}{\mathrm{RF} \times \mathrm{Area}_{\ \mathrm{internal\ standard}}} \times 100\%$$

The cyclohexane oxidation reaction was used as an example to obtain its yield by GC: Cyclohexanone (0.2 mmol) and 1,3,5-trimethoxybenzene (0.2 mmol) were dissolved in acetonitrile (2 ml) as the standard sample for GC testing, and calculating the RF of cyclohexanone.

Figure S26. GC data of standard sample with cyclohexanone (0.2 mmol) and 1,3,5-trimethoxybenzene (0.2 mmol).

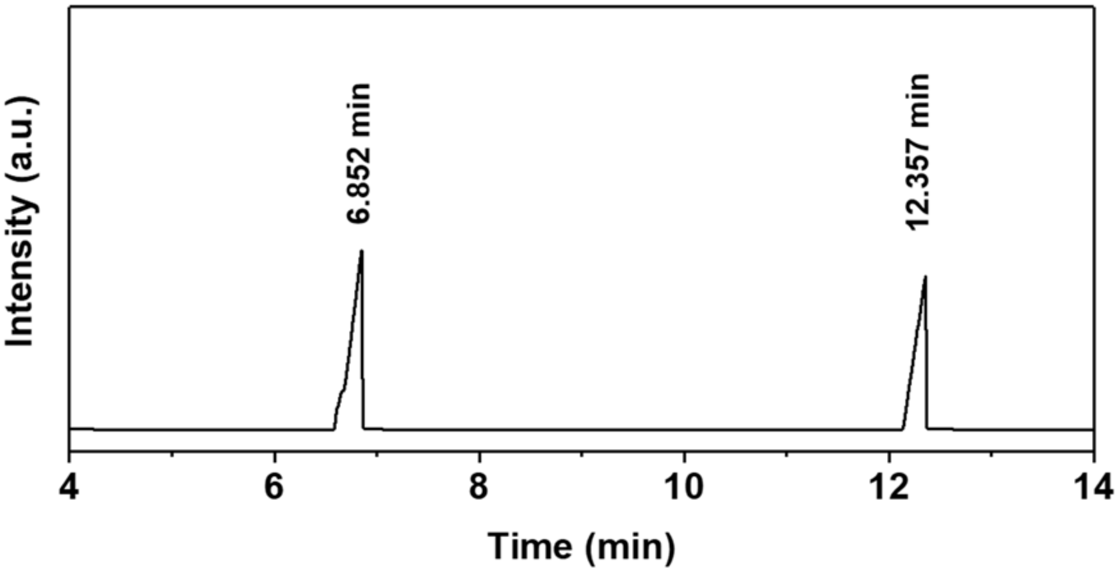

Peak	Retention Time (min)	Area (%)
1	6.852	100
2	12.357	79.7

$$\mathrm{RF} = \frac{\mathrm{Area}_{\ \mathrm{target\ product}}}{\mathrm{Area}_{\ \mathrm{internal\ standard}}} = \frac{100}{79.7} = 1.25$$

The filtrate of the cyclohexane oxidation reaction was subjected to GC test after addition of 1,3,5-trimethoxybenzene (0.2 mmol) as an internal standard, and the yield was calculated from the area ratio.

Figure S27. GC data of crude cyclohexane oxidation reaction mixtures with 1,3,5-trimethoxybenzene (0.2 mmol) as the internal standard.

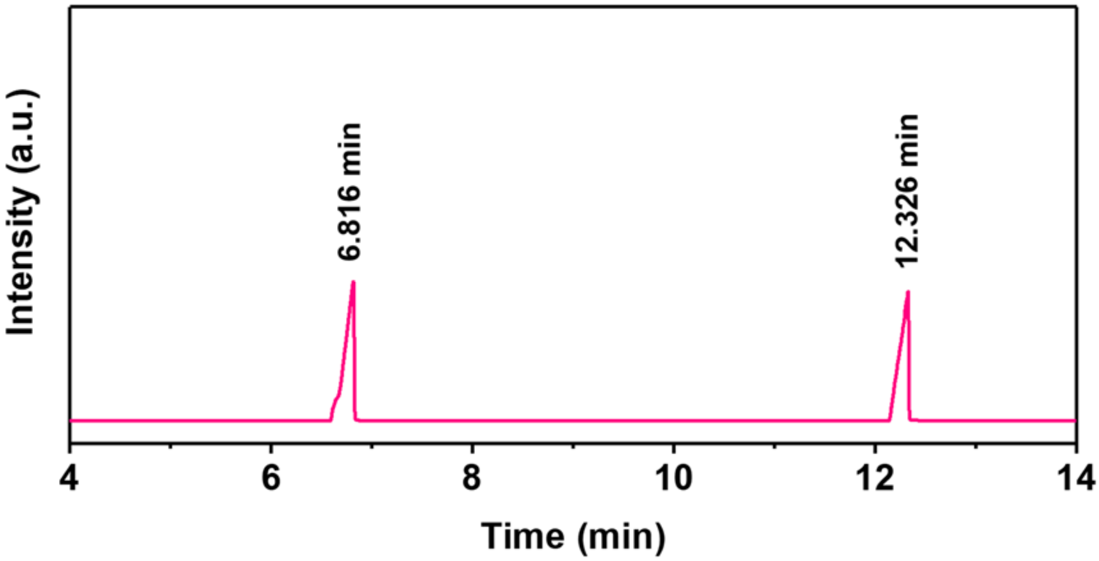

Peak	Retention Time (min)	Area (%)
1	6.816	100
2	12.326	93.5

$$\text{Yield (\%)} = \frac{\text{Area}_{\text{target product}}}{\text{RF} \times \text{Area}_{\text{internal standard}}} \times 100\% = \frac{100}{1.25 \times 93.5} \times 100\% = 85\%$$

10. References

1. Garai B., Mallick A.&Banerjee R. Photochromic metal–organic frameworks for inkless and erasable printing. *Chem. Sci.* **7**, 2195−2200 (2016).
2. SMART, Data collection software (version 5.629) (Bruker AXS Inc.; Madison, WI, 2003).
3. SAINT, Data reduction software (version 6.45) (Bruker AXS Inc.; Madison, WI, 2003).
4. Sheldrick, G. SHELXTL97, Program for Crystal Structure Solution (University of Göttingen: Göttingen, Germany, 1997).
5. Spek, A. L. Single-crystal structure validation with the program PLATON. *J. Appl. Cryst.* **36**, 7−13 (2003).
